# Ultracompact meta-imagers for arbitrary all-optical convolution

**DOI:** 10.1038/s41377-022-00752-5

**Published:** 2022-03-18

**Authors:** Weiwei Fu, Dong Zhao, Ziqin Li, Songde Liu, Chao Tian, Kun Huang

**Affiliations:** 1 Department of Optics and Optical Engineering, University of Science and Technology of China, Hefei, Anhui 230026 China; 2 Institute of Artificial Intelligence, Hefei Comprehensive National Science Center, Hefei, Anhui 230088 China; 3 Department of Precision Machinery and Precision Instrumentation, University of Science and Technology of China, Hefei, Anhui 230026 China

**Keywords:** Sub-wavelength optics, Metamaterials

## Abstract

Electronic digital convolutions could extract key features of objects for data processing and information identification in artificial intelligence, but they are time-cost and energy consumption due to the low response of electrons. Although massless photons enable high-speed and low-loss analog convolutions, two existing all-optical approaches including Fourier filtering and Green’s function have either limited functionality or bulky volume, thus restricting their applications in smart systems. Here, we report all-optical convolutional computing with a metasurface-singlet or -doublet imager, considered as the third approach, where its point spread function is modified arbitrarily via a complex-amplitude meta-modulator that enables functionality-unlimited kernels. Beyond one- and two-dimensional spatial differentiation, we demonstrate real-time, parallel, and analog convolutional processing of optical and biological specimens with challenging pepper-salt denoising and edge enhancement, which significantly enrich the toolkit of all-optical computing. Such meta-imager approach bridges multi-functionality and high-integration in all-optical convolutions, meanwhile possessing good architecture compatibility with digital convolutional neural networks.

## Abstract (web summary)

An ultra-compact metasurface-based imager with modified point spread function is demonstrated to realize arbitrary all-optical parallel picture convolution that is highly compatible to convolutional neural network.

## Graphical abstract

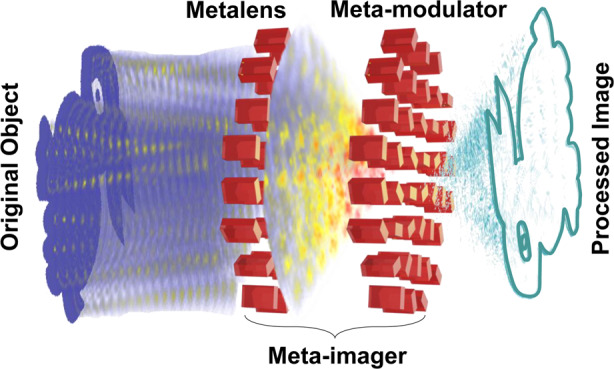

## Introduction

Artificial intelligence (AI) has recently gained rapid development in academies and industry due to intense research of deep convolutional neural networks (CNN) with a multilayer architecture^1^. In each layer, numerous convolutional operators with functionality-assigned kernels are implemented to extract important features of objects for identification, but they are extremely time-consuming with the increment of AI tasks^2^. Although advanced electronic devices such as graphics processing units^3^, field-programmable gate arrays^4^, and tensor processing unit^5^ have been proposed to accelerate the computation, the speed and energy consumption are still limited by the low response of electrons, such as charging and discharging in capacitance, electromagnetic radiation and create heat by the movement of the electrons in materials^6^.

In comparison, photons as massless bosons allow lossless propagation and manipulation of light through large-bandgap transparent materials for optical parallel analog computing without analog-to-digital and digital-to-analog convertors^7–9^, hereby enabling high-speed and low-consumption computation. Currently, all-optical convolutional computing has two main approaches^10^: Fourier spatial filtering^11–17^ and Green’s function (GF)^18–27^. The Fourier method employs a couple of lenses to realize spatial-frequency transform of original data for spatial-spectrum filtering via a modulation mask that is traditionally pure-phase or pure-amplitude, and inverse transforms for reconstructing processed data. Such a configuration with multiple elements is not preferred in integrated photonic systems, meanwhile, the complex filter with both amplitude and phase modulation is mandatorily needed for an arbitrary convolutional operation but unachievable for most traditional optical elements. The GF approaches implement optical analog computing by modulating angle-dependent transmittance (or reflection) with surface plasmon polaritons^18^, artificial nano-resonators^19^, photonic crystals^20^, metasurfaces^21–25^, topological photonics^26^, and spin Hall effect of light^27^. Although some GF approaches operating in a single device enable high integration, they have limited angular responses (usually valid for only one special operation), which are insufficient for arbitrary convolutional computing in AI and image processing. Since both all-optical approaches have the drawbacks of either low integration or limited functionality, wavelength-multiplexing technologies combining optical and electronic operations have recently been demonstrated to accelerate convolutional computing in neural networks^28,29^, exhibiting a significant enhancement in speed compared with electronic computing. However, the electronic parts in such optoelectronic systems still constrain the ultimate speed.

Here, we propose a compact meta-imager to realize all-optical convolutional computing with arbitrary kernels in a parallel and real-time way. This meta-imager contains two parts: a metalens for image formation and the other complex-amplitude meta-modulator for reshaping its point spread function (PSF), which can be highly integrated into a single meta-device. By correlating the convolutional operator of an arbitrary 3 × 3 matrix, we realize the corresponding complex-amplitude meta-modulator via geometric dielectric metasurfaces. We have successfully demonstrated multiple convolutional operations, such as spatial differentiation, denoising, edge detection, and enhancement, to improve the imaging quality of optical (phase- and amplitude-type) and biological samples (e.g., chromosome molecules, onion, and oral epidermal cells) with different magnifications and spatial resolutions. Such a combination of both multiple functionalities and compact volume is not possible in other approaches.

## Working principles of meta-imager

**Fig. 1:**
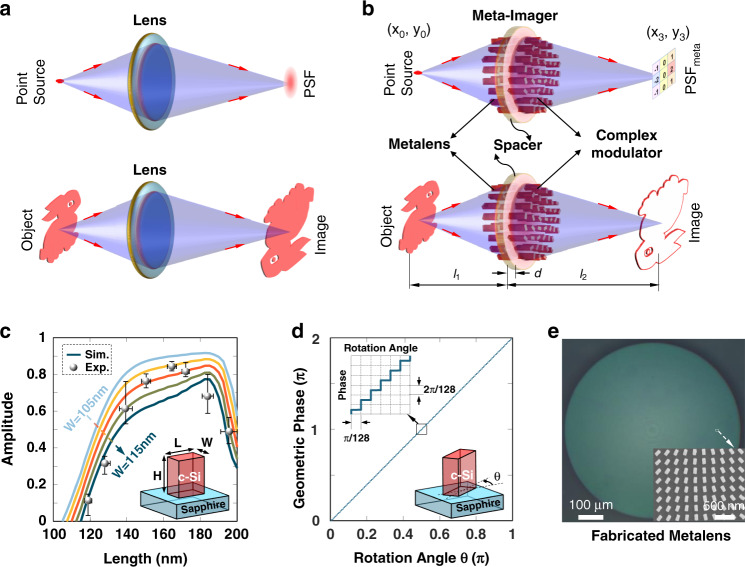
Working principle of meta-imager for arbitrary convolution operation. **a** Sketch for the imaging process of a single imaging lens with an Airy-spot-like point spread function (PSF). **b** Mechanism of meta-imager composed of a metalens and complex-amplitude meta-modulator. The PSF_meta_ of this meta-imager can be reshaped into an arbitrary pattern, which can be used as the kernel of the convolution operation. The spacer exists between the metalens and the meta-modulator. The position (*x*_0_, *y*_0_) and (*x*_3_, *y*_3_) stand for the coordinates of object and image spaces, respectively. **c** Simulated (curves) and experimental (dots) amplitude modulation by using geometric metasurfaces made of dielectric c-Si nanobricks with different lengths *L*, where the height *H* = 300 nm and the width *W* = 110 nm. Considering the fabrication error, we provide the simulated *L*-dependent amplitude profiles if the width *W* (from 105 nm to 115 nm) has deviated with a step size of ±2.5 nm. The simulation about the amplitude modulation is implemented in a finite-difference time-domain model with the periodic boundaries along *x* and *y* direction, and perfect-matching layers along *z*-direction. In our simulation, the periods (*p*_x_ and *p*_y_) of metasurfaces are taken to be 250 nm, which is below the operating wavelength (*λ* = 633 nm). The experimental amplitude (calculated as the square of polarization conversion efficiency) and length *L* (directly measured from the SEM images) of the nanobricks are derived from five individual samples, which exhibit similar behavior. The insert presents the configuration of a unit cell in geometric metasurfaces. **d** Phase modulation is determined by the rotation angle of nanobricks. One hundred twenty-eight-level phase modulation is employed to obtain a high-accuracy meta-modulator. The insert illustrates the orientation-rotated nanobricks with a rotating angle *θ*. **e** Optical and SEM (insert) images of our fabricated metalens, where the nanobriks have the dimension of *W* = 110 nm and *L* = 170 nm

Wave theory of light predicts image formation in a lens system as a convolutional operation between the optical field of an object and the PSF of the lens^30^ (Fig. 1a). With the PSF working as a kernel, the imaging system offers a natural choice for convolutional operation in a parallel, analog, and low-consumption way. However, the PSF in the imaging system usually has a fixed pattern such as Airy spot^31^, which cannot support arbitrary operation required in imaging processing and CNN.

To realize an arbitrary convolutional operation, we propose a meta-imager, composed of a metalens and a complex-amplitude meta-modulator (Fig. 1b), with a modified PSF. The metalens (with a focal length of *f*) and the meta-modulator (its complex amplitude denoted by *h*) are spaced by one-layer (e.g., substrate) or multilayer (e.g., air and substrate) transparent medium with an optical thickness of *d* = *n*_air_*t*_air_ + *n*_sub_*t*_sub_, where *n*_air/sub_ and *t*_air/sub_ are the refractive indices and the thicknesses of the air/substrate layer, respectively. To unveil the PSF of this meta-imager, a point source with its position (*x*_0_,*y*_0_) located at the object plane is assumed to illuminate the meta-imager. After a rigorous mathematical derivation (see Section 1 in Supplementary Materials), we have its pulse response at the image plane

1 $$\begin{array}{l}{\rm{PSF}}_{\mathrm{meta}}({x}_{3},{y}_{3};{x}_{0},{y}_{0})\propto {e}^{ik\frac{M}{2f}\cdot \frac{f-d}{{l}_{2}-d}\cdot {r}_{0}^{2}}\cdot\\ \left[\tilde{P}\left({x}_{3}+M{x}_{0},{y}_{3}\,+\,M{y}_{0}\right)\right]\otimes \left[F{(h)}_{{f}_{x}=\frac{{x}_{3}\,+\,M{x}_{0}}{\lambda \left({l}_{2}-d\right)},{f}_{y}\,=\,\frac{{y}_{3}+M{y}_{0}}{\lambda ({l}_{2}-d)}}\right]\end {array}$$

where the wavenumber *k* = 2*π*/*λ*, *λ* is the operating wavelength, *r*_0_^2^ = *x*_0_^2^ + *y*_0_^2^, *l*_1_ and *l*_2_ are the objects and image distances, the magnification *M* = *l*_2_/*l*_1_, $\tilde{P}$ is the PSF of the metalens without any modulator, *F*() stands for Fourier transform, the sign $\otimes$ is the convolution operation, *x*_3_ and *y*_3_ are the spatial coordinates at the image plane. In Eq. (1), the parabolic phase with the *r*_0_-position dependence introduces optical off-axis aberration such as coma and distortion, which, however, can be eliminated if *d* = *f*.

If an object has the electric field *U*(*x*_0_,*y*_0_), its coherent image can be expressed as^32^

2 $$\begin{array}{l}U({x}_{3},{y}_{3})=U({x}_{0},{y}_{0})\otimes {\rm{PSF}_{meta}}=U({x}_{0},{y}_{0})\\ \quad\,\qquad\qquad\otimes \tilde{P}\otimes F(h)=U{^{\prime}} ({x}_{3},{y}_{3})\otimes {\mathcal H}\end {array}$$

where $U{^{\prime}} ({x}_{3},{y}_{3})=U({x}_{0},{y}_{0})\otimes \tilde{P}$ is the image by the metalens without the modulator, and the item ${\mathcal H} =F(h)$ works as a convolutional operator. Equation (2) indicates that our meta-imager yields a convolutional operation between the magnified image and a spatial spectrum of the meta-modulator. Note that, only one lens is needed in this meta-imager, which therefore allows for lower cost and higher integration than the Fourier filtering system^11–17^. The spatial spectrum of the meta-modulator offers designable and customized kernels for various convolutional operations. *h* can be obtained by inverse Fourier transform of the expected operator, i.e., *h* = *F*^*−1*^(ℋ), where *F*^*−1*^() denotes the inverse Fourier transform and the convolutional operator ℋ of a 3 × 3 matrix is employed here for high compatibility with traditional image processing^33^ and CNN^2^. It, therefore, bridges the gap between the convolutional operator and the meta-modulator. Detailed instruction about getting the complex amplitude of the meta-modulator from a given matrix-type operator is provided in “Methods”.

To realize the complex-amplitude modulation, we utilize transmissive dielectric geometric metasurfaces^34–42^ composed of orientation-rotated nanobricks that could transfer circularly polarized incident light into its cross-polarized light with an additional phase of twice the rotation angle *θ*^41,42^. The conversion efficiency, related to the amplitude modulation, is determined by the dimension of the nanobricks. Since the rotation and the dimension of nanobricks can be manipulated separately (see Section 2 in Supplementary Materials), the phase and amplitude of the cross-polarized transmitted light are customized independently with a high spatial precision of subwavelength scale, hereby superior to traditional diffractive optical elements^43^, spatial light modulators^44^, and digital micromirror devices^45^. Experimentally, the dielectric geometric metasurfaces are demonstrated in a 300-nm thick (i.e., *H* = 300 nm) crystalline silicon (c-Si) film on a sapphire substrate. To facilitate the fabrication, the nanobricks have the fixed widths *W* = 110 nm, leaving the only variable (the length *L*) to modulate the amplitude.

Figure 1c presents the simulated and experimental amplitude profiles with a peak located around *L* = 165 nm, suggesting that both increasing and falling edges of this peak can be used to modulate the amplitude. At both edges, the amplitude changes quickly, which implies strong sensitivity to *L* and therefore needs high-quality fabrication. To release it, we utilize the discrete amplitude with three levels for doublet meta-imager (*d* ≠ 0) and five levels for singlet meta-imager (*d* = 0). Despite the error caused by this discretization of amplitude, simulated convolutions (see Section 3 in Supplementary Materials) are seldom influenced, since 128-level phase modulation (Fig. 1d) is employed here to compensate for the accuracy.

## All-optical convolution via a doublet meta-imager

For doublet meta-imager, the metalens and meta-modulator are fabricated separately in different specimens to facilitate their alignment, so that its realistic spacer contains both sapphire substrates of the metalens and meta-modulator and the in-between air. Its corresponding optical thickness *d* = *f* is used in this doublet meta-imager for reducing optical aberration. Figure 1d shows the fabricated metalens with good imaging and focusing functionalities, see the measurement details in Methods and Section 6 in Supplementary Materials.

### Edge detection

**Fig. 2:**
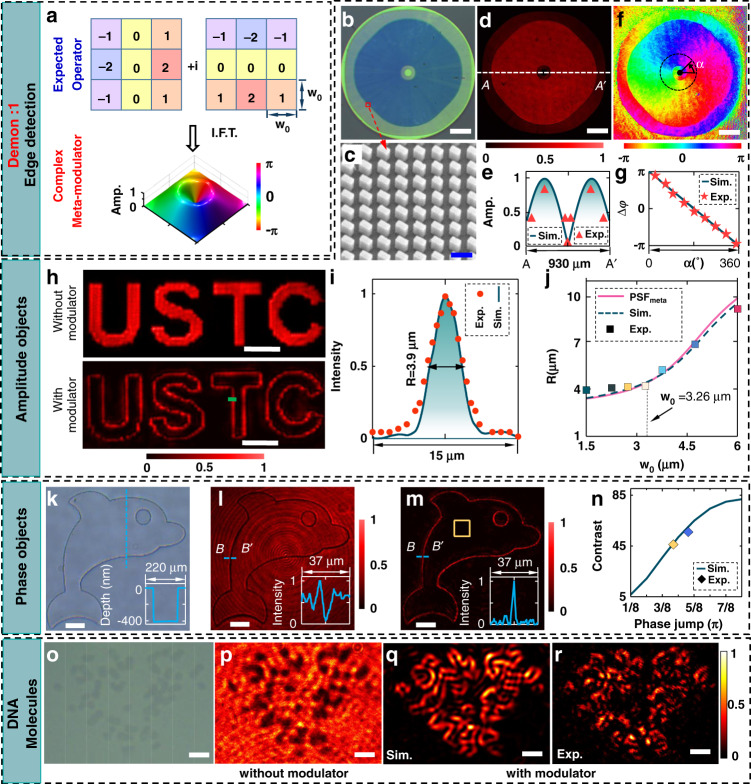
Edge detection. **a** Illustration of the expected operator (upper panel) for edge detection and its corresponding meta-modulator (lower panel) with amplitude and phase profiles. **b**, **c** Microscopic (**b**) and SEM (**c**) images of the fabricated meta-modulator with the corresponding *w*_0_ = 1.5 μm. The colors in the microscopic image (**b**) originate from the wavelength-dependent reflection of different nanobricks. Scale bars: 100 μm (**b**); 300 nm (**c**). **d** Transmission (cross-polarization part) of the fabricated meta-modulator under the circular-polarized illumination. Scale bar: 100 μm. **e** Simulated (curve) and experimental (triangles) line-scanning amplitude profiles along the line AA′ (denoted in (**d**)). The experimental amplitude is obtained by using a square root of the transmission in (**d**). **f** Retrieved phase profiles from the experimental interference patterns. The sign *α* denotes the azimuthal coordinate. Scale bar: 100 μm. **g** Simulated (curve) and experimental (star) phase profiles along the azimuthal coordinate *α*. The presented phase is defined as ∆*φ* = *φ*(*α*) − *φ*(0), which is used to remove the constant phase. **h** Measured images by a single metalens (without meta-modulator, upper panel) and the meta-imager (with meta-modulator, lower panel). Scale bars: 50 μm. **i** Simulated (curve) and measured (dots) line-scanning intensity profiles along the green line in (**h**). The processed edges have the full width at half maximum (FWHM): *R* = 3.9 μm. **j** Relationship between the widths of processed edges and the detection accuracy (*w*_0_) of the ideal modulator. Both simulated (dashed) and experimental (squares) results have good agreement with the predicted ones (solid curve) by using Eq. (1). The demonstrated metalens has the PSF with the size of 3.3 μm, which determines the best resolving power of the proposed meta-imager. **k** Microscopic image of a dolphin pattern etched on a quartz substrate. The etching depth is around 400 nm (insert), which is measured by using a profilometer. Scale bar: 50 μm. **l** Transmitted image of this binary-phase dolphin. The dark intensity is caused by the phase discontinuity at the edge. The line-scanning intensity across the dark edge is plotted in the insert. Scale bar: 50 μm. **m** Processed image by using the meta-imager at the magnification *M* = 1. In the insert, the line-scanning intensity along the line BB′ shows the clear edge that has a high contrast to the surrounding background. Scale bar: 50 μm. **n** Dependence of the contrast (defined as the ratio of the intensity at the edge to the background intensity) on the phase jump of the binary-phase object. To make a fair comparison with the simulated contrast (curve), we remove the background noise (caused by the transmitted co-polarization light) when calculating the experimental (diamond) contrast. The experimental background intensity is evaluated by using the average intensity encircled within the yellow square in (**m**), where the intensity is less influenced by the diffraction of extracted edges due to the large distance from the edge. **o** Reflective microscopic image of DNA molecules. Scale bar: 5 μm. **p**–**r** Outlining the edge of the DNA molecules. To match the resolution of our meta-imager, these DNA molecules undergo a magnification of 4 by using an objective lens. The magnified DNA molecules (**p**) are just located at the input plane of our meta-imager (*M* = 1), which outputs the edges (see **q** in simulation and **r** in the experiment) of the DNA molecules at the image plane. Scale bars: 20 μm

Since Eq. (1) allows arbitrary convolution operator ℋ, we realize edge detection of an object by using a complex-valued operator composed of two orthogonal (*x* and *y* direction) differentiation (see Fig. 2a)

3 $${ {\mathcal H} }_{ED}={ {\mathcal H} }_{x}+i{ {\mathcal H} }_{y}=\left[\begin{array}{ccc}-1 & 0 & 1\\ -2 & 0 & 2\\ -1 & 0 & 1\end{array}\right]+i\,\left[\begin{array}{ccc}-1 & -2 & -1\\ 0 & 0 & 0\\ 1 & 2 & 1\end{array}\right]$$

where the real part ℋ_*x*_ and the imagery part ℋ_*y*_ denote the *x*- and *y*-direction operators^33^, respectively. In Eq. (3), every matrix element has the spatial pitch of *w*_0_ × *w*_0_, which defines the detection accuracy of the convolutional operator. After substituting Eq. (3) into Eq. (2), we have *I* = *U*′_*x*_^2^ + *U*′_*y*_^2^ (where *U*′_*x*_ = *U*′⨂ℋ_*x*_ and *U*′_*y*_ = *U*′⨂ℋ_*y*_ denote the *x-* and *y*-direction differentiations, respectively), which therefore reveals the edge details of the original image *U*′. By implementing the inverse Fourier transform of ℋ_*ED*_, we obtain the complex amplitude of the meta-modulator, which has a helical phase and a doughnut-shape amplitude (Fig. 2a). For the edge-detection meta-imager, these detailed analyses result naturally in the azimuthal phase of exp(*iφ*), which is also required in spiral phase contrast microscopy (see Section 14 in Supplementary Materials)^46–48^. However, our meta-imager needs an additional amplitude modulation that can be used to control the detection accuracy (as shown later). The fabricated meta-modulator has been characterized (Fig. 2b–g) and discussed in more detail in Methods.

Firstly, we detect the edge of an amplitude object (“USTC” etched through a chromium film, see Fig. 2h) in a self-made optical system (see Section 8 in Supplementary Materials). Our meta-imager yields the clear edge of “USTC” (lower panel), with the magnification *M* = 1. Other larger-magnification edges by using the same meta-imager are also obtained without the loss of detection quality (see Section 9 in Supplementary Materials). The uniform edges with high contrast to the background are superior to others reported results^7,9^, implying an efficient edge detection. The realistic detection accuracy, evaluated by the width of the outputted edge, is determined by the mutual interplay between the PSF of the metalens and the spatial pitch *w*_0_ of the convolutional operator (as indicated in Eqs. (1) and (2)). Figure 2i shows the achieved accuracy of *R* = 3.9 μm at *M* = 1 and *w*_0_ = 1.5 μm, with high consistency between simulation and experiment. To unveil its dependence on *w*_0_, we have fabricated the meta-modulators with different *w*_0_ and measured the accuracy (see Fig. 2j) at *M* = 1. When *w*_0_ is larger than the spot size (denoted by *r*_0_ = 3.26 μm at *M* = 1) of the metalens’ PSF, the realistic accuracy exhibits a quasi-linear dependence on *w*_0_. But, when *w*_0_ < *r*_0_, the PSF of the metalens dominates the achieved accuracy, which is nearly constant for a given metalens. Therefore, the detection accuracy in our meta-imager is determined by the larger one between *w*_0_ and *r*_0_. A higher-NA metalens can enhance the detection accuracy but at the cost of the decreased efficiency (caused by angle-dependent polarization conversion^41^) and shrunken field-of-view (induced by the increment of optical aberration^31^). Our current meta-imager enables edge detection with a field-of-view better than 710 μm × 710 μm at *M* = 1 (see Section 10 in Supplementary Materials).

This meta-imager can also extract the edge of a pure-phase object. Figure 2k shows an optical microscope image of a transparent dolphin with an etched depth of 400 nm on a quartz substrate, leaving a phase jump of 0.577*π* at *λ* = 633 nm. Under the illumination, this binary-phase dolphin has a uniform transmission (Fig. 2l) over the entire field of view, except at the dark boundary caused by the phase discontinuity. When this dolphin works as the input object, our meta-imager highlights only the edge but suppresses the background (Fig. 2m), implying the better identification of the object. The contrast of the identified edge to the background is maximum (the best identification) for the phase jump of (2*n* + 1)*π* but minimum (no identification) for the phase jump of 2*nπ*, where *n* is an integer. Experimentally, we have checked two-phase jumps of 0.433*π* (300-nm depth) and 0.577*π* (400-nm depth), which yield well-consistent contrasts with the simulations (see Fig. 2h).

In addition, the meta-imager is employed further to detect the edges of chromosome molecules. The microscopic reflective (Fig. 2o) and magnified transmissive (Fig. 2p) images of weakly dyed chromosome molecules with both amplitude and phase show either low contrast or blurred edges, which is insufficient for distinguishing these chromosomes molecules. In comparison, our meta-imager outlines these chromosome molecules by highlighting the edges in both simulation (Fig. 2q) and experiment (Fig. 2r). The inhomogeneity of the experimental edges originates mainly from optical misalignment between the metalens and the meta-modulator, meanwhile, the unresolved small gaps between two neighboring molecules also weaken the edges.

Note that, all the demonstrations for edge detection are achieved in parallel at the speed of light, leaving the processing time of ~10^−11^ s (evaluated by the ratio of the optical path to the speed of light), enhanced by 9 orders of magnitude compared with electronic digital convolutions (at the level of ~10^−2^ s, estimated by implementing a 2-dimensional convolution between two 100 × 100 matrices with MATLAB software in a personal computer (CPU: Intel Core I7-7500U)). It, therefore, enables real-time edge detection of a running “horse” in a movie (Movies 1 and 2). To simulate high-speed moving objects in a laboratory environment, we have loaded the “horse-running” video onto an amplitude-type spatial light modulator (SLM, Holoeye) with a refresh frequency of 60 Hz. The SLM is located at the input plane of our doublet meta-imager with the parameters *M* = 1 and *w*_0_ = 1.5 μm. Thus, the “horse-running” video is taken as the object. Correspondingly, the detected results at the output plane are captured in real-time by our camera (Thorlabs). The real-time recording of the detected “running-horse” is provided in Supplementary Video 2, while Supplementary Video 1, as a control case, shows the real-time “running-horse” without the meta-modulator.

Moreover, edge enhancement has also been demonstrated by using a real-value operator

4 $${ {\mathcal H} }_{EE}=\left[\begin{array}{ccc}-1 & -1 & -1\\ -1 & 12 & -1\\ -1 & -1 & -1\end{array}\right]$$

which leads to a complex meta-modulator with saddle-shaped amplitude and linear phase (Fig. S10). Since edge enhancement resembles closely edge detection, its detailed discussions are provided in Section 11 in Supplementary Materials, where the good agreement between simulated and experimental results confirms its validity.

### Spatial differentiation

**Fig. 3:**
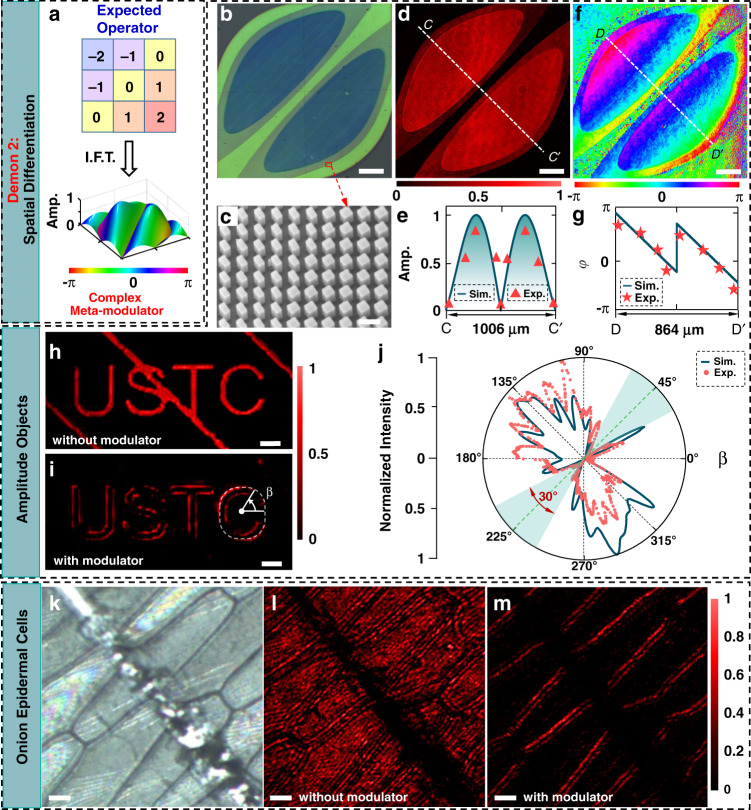
Spatial differentiation. **a** Proposed operator (upper panel) and its related meta-modulator (low panel) for 135°-direction spatial differentiation. In the panel of meta-modulator, the pseudo-color denotes the phase. **b**, **c** Microscopic (**b**) and SEM (**c**) images of the fabricated meta-modulator with the corresponding *w*_0_ = 1.5 μm. The nanobricks having the same dimension as the edge-detection case are employed to realize three-level amplitude modulation. Scale bars: 100 μm (**b**); 300 nm (**c**). **d** Transmission (cross-polarization part) of the fabricated meta-modulator under the circular-polarized illumination. Scale bar: 100 μm. **e** Simulated (curve) and experimental (triangles) line-scanning amplitude profiles along the line *CC*′ (denoted in (**d**)). The experimental amplitude are obtained by using a square root of the transmission in (**d**). **f** Retrieved phase profiles from the experimental interference patterns. Scale bar: 100 μm. **g** Simulated (curve) and experimental (stars) phase profiles along the line *DD*′ (denoted in (**f**)). **h** Original object was obtained by using a single metalens without the modulator. Scale bar: 20 μm. **i** Processed the image by our meta-imager with the modulator. The 135°-direction lines in (**h**) are removed by the designed spatial differentiator. To test the directionality of this spatial differentiator, the azimuthal coordinate *β* is sketched along the outmost edge (shown in a white-dashed curve) of the processed character “C”. Scalebar: 20 μm. **j** Simulated (curve) and measured (dots) intensity profiles of “C” pattern at the azimuthal coordinate *β* (sketched in (**i**)). The edge of the character “C” is removed around the 45°–225° line with a range of 30°. **k**–**m** Microscopic image (**k**) of onion epidermal cells contaminated by an opaque needle. After being imaged (with a magnification of 1.5) by an objective lens, the magnified cells (**l**) immediately operate as the input of our meta-imager (*M* = 1), which creates the edges (**m**) of these onion cells at the image plane. Scale bars: 20 μm in (**k**); 30 μm in (**l**, **m**)

One-dimensional spatial differentiation is important to extract or remove directional details in imaging and data processing^33^. As an example, a meta-modulator realizing 135°-direction differentiation is proposed by linking a convolutional operator

5 $${ {\mathcal H} }_{SD}=\left[\begin{array}{ccc}-2 & -1 & 0\\ -1 & 0 & 1\\ 0 & 1 & 2\end{array}\right]$$

which works as a directional derivative filter. Its corresponding meta-modulator (see the microscopic image in Fig. 3b and SEM image in Fig. 3c) has the expected amplitude and phase modulation (see Fig. 3d–g and Methods).

To validate its performance, a binary-amplitude object with 135°-direction line-shape defects (Fig. 3h) is used as the input of this differentiation meta-imager. Figure 3i shows the outputted results, where the defects are removed completely for better identification of “USTC”. Additionally, this differentiator also yields the edges of objects along the designed direction (135°–315°) but eliminates the edges along the orthogonal direction (45°–225°). The azimuthal intensity profiles of the processed “C” are illustrated in Fig. 3j, showing a cancellation range of 30° near the 45°–225° direction. It implies that the 5/6 contour of an object can be detected by using this differentiator.

Furthermore, this differentiator is employed to filter out undesired directional defects in onion epidermal cells (Fig. 3k). The transmitted patterns (Fig. 3l) of the epidermal cells show line-shape darkness (where the opaque defect lies) with the blurred and unresolved boundaries between the two cells. In Fig. 3m, our meta-imager creates the defect-free long-axis edges of the cells, meanwhile eliminating short-axis edges that are nearly parallel to the differentiation direction. Experimentally, the short-axis edges can be extracted via rotating the cells or the meta-modulator by 90°.

### Denoising

**Fig. 4:**
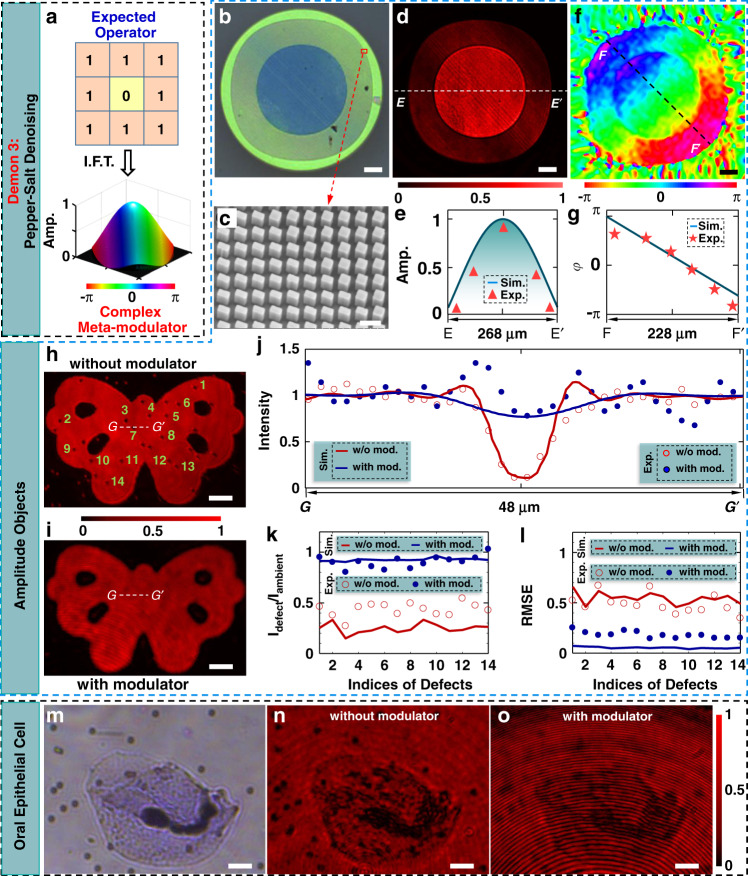
Pepper-salt denoising. **a** Proposed operator (upper panel) and its corresponding meta-modulator for pepper-salt denoising. The size *w*_0_ of every element in this operator is designed to be the size of defects. Thus, after processing by the meta-imager for salt denoising, the intensity profiles at the defects are taken as the averaged intensity at the surrounding positions, hereby removing the defects. **b**, **c** Microscopy (**b**) and SEM (**c**) images of our fabricated meta-modulator for pepper-salt denoising. Scale bars: 20 μm in (**b**); 300 nm in (**c**). **d** Transmitted (cross-polarized part) pattern of the meta-modulator under the circular-polarization illumination. Scale bar: 20 μm. **e** Simulated (curve) and experimental (triangles) line-scanning amplitude profiles along the line *EE*′ (denoted in (**d**)). The experimental amplitude is obtained by using a square root of the transmission in (**d**). **f** Retrieved phase profiles from the experimental interference patterns. Scale bar: 20 μm. **g** Simulated (curve) and experimental (stars) phase profiles along the line *DD*′ (denoted in (**f**)). **h** Transmitted pattern of a binary-amplitude butterfly (made in an opaque chromium film on a quartz substrate) that is captured by using a single metalens without the modulator. Some of the defects are indexed as examples in the following characterizations. Scale bar: 20 μm. **i** Processed an image of a butterfly that is located at the input plane of our denoising meta-imager. Scale bar: 20 μm. **j** Normalized intensity profiles along the line GG′ (sketched in (**h**, **i**)). Each intensity profile is normalized to the averaged intensity outside the defect 7. **k**, **l** Simulated (curves) and experimental (dots and circles) intensity ratios (**k**) and RMSEs (**l**) at 14 exemplified defects. The data before (w/o mod.) and after (with mod.) using the meta-modulator is compared in detail to observe the improved uniformity. Without introducing the error, we only examine the uniformity of intensity at a defect-centered square of 12 μm × 12 μm (i.e., the efficient area of the operator). In **k**, the averaged intensity at the defect region (i.e., 4 μm × 4 μm) is labeled as *I*_defect_, while the averaged intensity at the left region of the square is calculated as *I*_ambient_. **m** Microscopic image of an oral epithelial cell with random defects. The defects have a size of ~2 μm × 2 μm. Scale bar: 10 μm. **n** A 2.4-fold magnified pattern of the oral epithelial cell. The magnified defects have the size of 4.8 μm × 4.8 μm and work immediately as the input of our denoising meta-imager. Scale bar: 24 μm. **o** Processed image by using our meta-imager. Scale bar: 24 μm

Pepper-salt noise refers to randomly distributed defects existing in various imaging systems and is removed usually by the median filtering method in digital data processing^33^. However, to the best of our knowledge, its all-optical solution to pepper-salt denoising has not to be reported yet due to the limited functionalities in the previous approaches^7,9^. By linking the electronic convolutional operators and all-optical meta-modulators straightforwardly, our meta-imager suggests one solution to all-optical pepper-salt denoising. Here, we propose a convolutional operator

6 $${ {\mathcal H} }_{\rm{PSDN}}=\left[\begin{array}{ccc}1 & 1 & 1\\ 1 & 0 & 1\\ 1 & 1 & 1\end{array}\right]$$

which removes the random noise by the averaged intensity at the surrounding eight positions of the defect. The resulting meta-modulator has been fabricated (see the microscopic image in Fig. 4b and SEM image in Fig. 4c) in high quality, exhibiting well-performed phase and amplitude (see Fig. 4d–g and more details in Methods).

Figure 4h shows the image of a butterfly pattern with random defects having the size of 4 μm × 4 μm. By employing the denoising meta-imager (with a well-matched accuracy *w*_0_ = 4 μm), we obtain the processed image with much-enhanced uniformity (Fig. 4i), where the dark defects have been removed efficiently with nearly equal intensity to their surroundings. Figure 4j illustrates the line (GG′ in Fig. 4j)-scanning intensity profiles across the exemplified defect. In contrast to the non-denoised (without modulator) case, the denoising meta-imager enhances the valley (minimum) intensity within the defect by ~6 times. The denoised intensity around the defects has a slight variation (see the blue dots in Fig. 4j) caused by the interference with the co-polarized background, which, however, can be suppressed by using a higher-extinction polarization analyzer. Nevertheless, the ratios (Fig. 4k) of the averaged intensity within the defect to the ambient intensity approach are 0.9, which is enhanced by 2.25 times compared to ~0.4 for the non-denoised case, indicating the improved homogeneity. To quantify the uniformity, the root-mean-square error (RMSE) between the normalized intensity around the defect and the ideal flat intensity is employed and shown in Fig. 4l. The RMSE of ~0.5 for the non-denoised case is suppressed down to ~0.18 after denoising, implying a ~2.78-fold improvement in uniformity. Both ratios and RMSEs have suggested an enhancement factor of >2, hereby confirming the denoising ability.

We have also applied this denoising meta-imager to remove random defects in oral epithelial cells (Fig. 4m). These cells have the transmitted pattern (Fig. 4n) with dark dots caused by these opaque defects. After being magnified by an objective lens, these cells are processed by the denoising meta-imager. Despite the existence of the co-polarized background, the intensity within the defect is observed with improved homogeneity (Fig. 4o). The un-eliminated darkness at the defect region originates from the magnified defects, which leads to the mismatch between the size (i.e., 4.8 μm in Fig. 4n) of magnified defects and the accuracy (*w*_0_ = 4 μm) of the operator. Our simulation shows that the meta-imager behaves well when the defect has a size below *w*_0_ (see Section 12 in Supplementary Materials).

## All-optical convolution via a singlet meta-imager

**Fig. 5:**
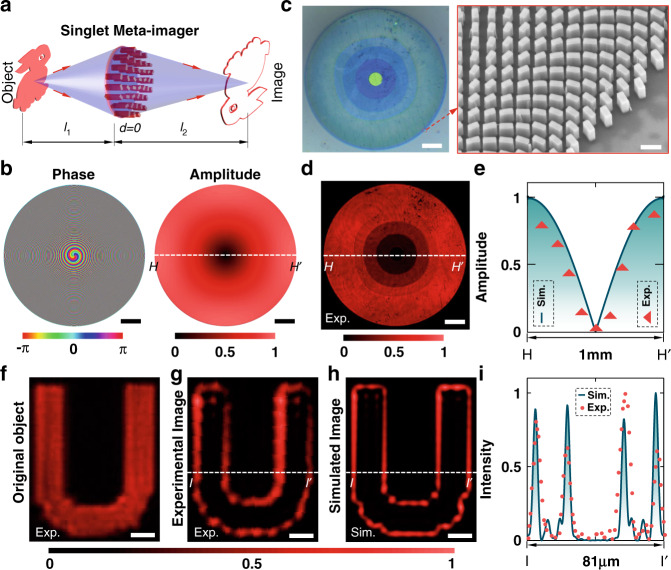
Singlet meta-imager for edge detection. **a** Sketch for singlet meta-imager with the object and image distances labeled in *l*_1_ and *l*_2_, respectively. **b** Phase and amplitude profiles of the meta-imager realizing the edge detection. Its phase is obtained by adding both phases of metalens (*f* = 2.5 mm) and meta-modulator. The amplitude modulation comes from only the meta-modulator part because the metalens has an amplitude of 1. Scale bars: 100 μm. **c** Microscopic (left) and SEM (right) images of our fabricated singlet meta-imager with five discretized amplitudes. These amplitude profiles of 1, 0.75, 0.55, 0.3, and 0 are realized by using the nanobricks with (*W* = 110 nm, *L* = 160 nm), (*W* = 110 nm, *L* = 150 nm), (*W* = 110 nm, *L* = 140 nm), (*W* = 110 nm, *L* = 130 nm) and (*W* = 150 nm, *L* = 150 nm), respectively. Scale bars: 100 μm (left panel) and 300 nm (right panel). **d** Transmitted (cross-polarized part) pattern of the singlet meta-imager under the circular-polarization illumination. Scale bar: 100 μm. **e** Experimental (triangles) and simulated (curves) amplitude profiles along the line *HH*′ (see (**b**, **d**)). The experimental amplitude profiles are extracted from the measured transmission. **f**–**h** Characterization of edge detection by using the singlet meta-imager. The original binary-amplitude object **f** is located at the input plane of the singlet meta-imager, which yields the experimental (**g**) and simulated (**h**) edges with *M* = 1. Scale bars: 10 μm. **i** Experimental (dots) and simulated (curves) intensity profiles along the line *II*′ (see (**g**, **h**))

After setting *d* = 0, the metalens and the meta-modulator are combined into a single complex-amplitude meta-device (Fig. 5a) that possesses both functionalities of imaging and modulator simultaneously, thus enabling high integration. However, the cost is the shrunken field-of-view, which is determined by the *d*-dependent phase item in Eq. (1). In the current configuration (i.e., *M* = 1, *f* = 2.5 mm), our simulation predicts that the convolution works well when the parabolic phase $k\frac{M}{2f}\cdot \frac{f-d}{{l}_{2}-d}{r}_{0}^{2} \,<\, 0.8\pi$ (see Section 13 in Supplementary Materials), suggesting a field-of-view of *r*_0_ = 50 μm for this singlet meta-imager.

We fabricate the singlet meta-imager (see the amplitude and phase profiles in Fig. 5b) that exhibits excellent performance (see Fig. 5c–e and more details in Methods). Then, we implement edge detection of a binary-amplitude object “U” (Fig. 5f) with this singlet meta-imager. Both experimental (Fig. 5g) and simulated (Fig. 5h) images reveal the clear edges with good agreement, as doubly confirmed by the line-scanning intensity with well-coincided widths and locations of the edges (Fig. 5i). The distance of ~94 μm between the top and bottom edges presents the experimentally achieved field-of-view, which approaches the simulated 100 μm.

## Discussions

The changeable distance *d* between the metalens and the meta-modulator enables our meta-imager to realize complex-amplitude manipulation at the coordinate space, which is fundamentally distinguished from the Fourier filtering approach operating at the frequency space^11–17^. The resulting advantage is the more compact volume of the entire device via singlet or doublet meta-surfaces. Particularly, the metalens and the meta-modulator can be made on the front and back sides of a substrate^49^, leading to the nearly identical volume as singlet meta-imager and maintaining the field-of-view simultaneously. For a given meta-imager, the outputted convolutional results can be magnified or shrunken on demand, which is more flexible to match the subsequent detection systems than previous approaches. A detailed comparison among them is provided in Table 1 in Supplementary Materials, suggesting that our meta-imager has superior performances, such as arbitrary convolutional operation, high integration, tunable magnification, and high accuracy. For light with other states of polarization, the complex amplitude in our meta-imager might be realized by using a pure-amplitude or pure-phase spatial light modulator with carefully designed encoding technique^50,51^. However, it will lead to increased volume, decreased efficiency, low spatial resolution, and low detection quality.

In summary, we have reported a meta-imager approach to realize all-optical convolutional operation with unlimited kernels. By modifying the PSF via the meta-modulator, the convolution between an object and the improved PSF can be simplified into straightforward imaging formation, which yields the expected processing pattern at the imaging plane. Our meta-imager allows at most two elements (i.e., imaging and modulating parts), both of which can be integrated into a single device for a compact volume. Some frequently used convolutional operations have been demonstrated with good performance to enhance the quality of images in optics and biology, which can be extended to artificial intelligence and high-performance computing.

## Materials and methods

### Obtaining the meta-modulator from a given convolutional operator

Equation (1) has shown the straightforward Fourier relationship between the meta-modulator (with the complex amplitude *h*) and the convolutional operator (ℋ). In practical applications, the operator ℋ in different formats (e.g., a discrete *N* × *N* matrix or an analytical formula) is usually known with specialized functionality. To obtain its corresponding meta-modulator numerically, we put the operator ℋ at the front focal plane of a Fourier lens with a focal length of *l*_2_–*d*. Thus, the Fourier transform of ℋ can be obtained at the rear focal plane, where the electric field is expressed as ${F}( {\mathcal H} )={F}({F}[h(x,y)])=h(-x,-y)$. Note that, *h*(−*x*,−*y*) and *h*(*x*,*y*) are centrosymmetric so that we can get *h*(*x*,*y*) via the symmetric transformation of *h*(−*x*,−*y*). During the numerical simulation, the sampling intervals (i.e., the pixel pitches) at both front and rear focal planes must be identical. For example, our meta-modulator has the pixel pitch of *p*_*x*_ × *p*_*y*_ (*p*_*x*_ = *p*_*y*_ = *p* = 250 nm), which should also be adopted in the convolutional operator ℋ. Because *w*_0_ in the operator ℋ is usually larger than the pixel pitch of the meta-modulator, one detection unit (i.e., *w*_0_ × *w*_0_) in ℋ contains the pixels of *P* × *P* (*P* = *w*_0_/*p* must be an integer), indicating an upsampling process. Then, the zeros are padded symmetrically around the upsampling ℋ to keep the same matrix size with the meta-modulator, as required in the numerical calculation. Note that, if the operator ℋ has a larger-size matrix of *N* × *N* (*N* > 3), the same process with the case of *N* = 3 is needed to obtain its corresponding meta-modulator. But, due to the non-zero matrix elements at the volumes or rows of *N* > 3, the larger-size matrix usually increases the efficient *w*_0_, hereby decreasing the detection accuracy. Therefore, such a larger-size matrix ℋ is usually not recommended in practical applications unless the 3 × 3 matrix fails to realize the expected functionalities. If the operator ℋ has an analytical form, it should also be digitalized with the sampling pixel pitch of *p*_*x*_ × *p*_*y*_.

Once the upsampling ℋ with the symmetrically padded zeros is well-prepared, we put it at the front focal plane of the Fourier lens, without any deviation (i.e., *x*_0_ = 0, *y*_0_ = 0). Thus, according to Fourier optics^52^, the ℋ has the diffraction field at the rear focal plane, which is taken as the complex amplitude *h*(*−x*,*−y*) of the meta-modulator. All the simulations about this diffraction process are implemented by using the Rayleigh–Sommerfeld integral^31^.

### The optical performance of fabricated metalens

To maximize the efficiency, we utilize a 128-level phase-type metalens with *f* = 2.5 mm and a diameter of 1 mm, which are chosen after a careful balance between the thickness of the substrate, the imaging resolution of the metalens^31^, and the angle-dependent conversion efficiency^41^ of the nanobricks. Figure 1e shows optical and SEM images of our fabricated metalens (see the fabrication details in Section 4 in Supplementary Materials) with a measured efficiency of 71.4% (see Section 5 in Supplementary Materials) at *λ* = 633 nm, which is highly consistent with the simulated amplitude (a square root of the efficiency) in Fig. 1c. The imaging and focusing functionalities of these metalens have also been verified experimentally with good performance (see Section 6 in Supplementary Materials), thus guaranteeing convolutional operations.

### Characterizing phase and amplitude from meta-modulators in doublet meta-imagers

For easy fabrication, all the meta-modulators of doublet meta-imagers in this work have discretized amplitude with three levels: 1, 0.5, and 0, which are realized by using the nanobricks with the dimensions (*W* = 110 nm, *L* = 170 nm), (*W* = 110 nm, *L* = 190 nm) and (*W* = 150 nm, *L* = 150 nm), respectively. The nano-bricks with the dimension of *W* = 150 nm and *L* = 150 nm are used here to facilitate the fabrication.

#### Edge-detection meta-modulator

After the discretization, the edge-detection meta-modulator (derived from the operator ℋ_*ED*_ with *w*_0_ = 1.5 μm) with the 128-level phase is fabricated in high quality, as confirmed from both optical (Fig. 2b) and SEM (Fig. 2c) images. Its performance is tested experimentally under the illumination of a circularly polarized beam, yielding the expected doughnut-contour transmission (Fig. 2d) with crossed polarization. Figure 2e shows the good agreement between experimental and simulated line-scanning amplitude profiles, implying the valid amplitude modulation. To characterize the phase modulation, a self-built Mach–Zehnder setup (see Section 7 in Supplementary Materials) interfering with the cross-polarized part of transmitted light with a slightly tilted co-propagating plane wave is used to generate an interference pattern, from which we can retrieve the experimental phase by using fast Fourier transform^53,54^. Figure 2f presents the retrieved vortex-like phase, which has a linear dependence on the azimuthal coordinate as observed in Fig. 2g and therefore verifies the creation of the required helical phase. Note that, a small phase jump at the boundaries between two size-different nanobricks occurs due to the propagation phase^55,56^, which has little influence on the entire performance of the meta-modulator and can be eliminated by adding the geometric phase with an equal and sign-opposite initial value.

#### Differentiation meta-modulator

For the differentiation operator, the element pitch of ℋ_SD_ is taken as *w*_0_ = 1.5 μm for the purpose of demonstration. Experimentally, the corresponding meta-modulator (see its microscopy image in Fig. 3b and SEM image in Fig. 3c) with 3-level amplitude and 128-level phase has a two-lobe-like transmission profile for the cross-polarization part (Fig. 3d). Along with the 135°-direction CC′ in Fig. 3d, the line-scanning amplitude profiles (Fig. 3e) have two peaks, which are sandwiched by three zeros at the center and both outmost terminals. Between both peaks, the amplitude exhibits the required quasi-linear dependence on the spatial frequency (referring to *k*_*x*_ = *x*/*fλ* due to *d* = *f*, where *x* is the spatial coordinate of meta-modulator and *f* is the focal length of the metalens) for spatial differentiation. Thus, from the position (*x*_*p*_ ≈ ±0.25 mm) of both peaks, one can evaluate the best differentiation accuracy of this meta-imager by using 0.5/*k*_*x*_ ≈ 3.17 μm, which is highly consistent with the PSF size (*r*_0_ = 3.26 μm) of the metalens. It implies that the larger-size meta-modulator (e.g., the outmost part beyond both peaks) is not necessary to enhance the accuracy (inherently determined by the PSF of the entire imaging system), which coincides with the predicted accuracy in Fig. 2j.

The retrieved phase from the interference pattern reveals a phase shift of *π* between two different lobes (see Fig. 3f), where the unstable phase at the zero-transmission region is caused by the oscillation of experimental noise. The good agreement between the experimental and simulated line-scanning phase profiles in Fig. 3g suggests valid phase modulation in this meta-modulator.

#### Denoising meta-modulator

The operator in Eq. (6) leads to a meta-modulator with a Gaussian-like amplitude and an additional linear phase (Fig. 4a), which is fundamentally distinguished from conventional low-pass filters with only the amplitude modulation^52^. The experimental meta-modulator (see its microscopic reflective image in Fig. 4b) has the ring-shaped pattern after the discretization of the amplitude in terms of three different nanobricks (Fig. 4c), thereby leading to the expected transmission (equivalent to amplitude modulation) as depicted in Fig. 4d. The quantitative comparison of the experimental and simulated amplitude profiles in Fig. 4e reveals their good consistency and confirms the validity of the fabricated meta-modulator. In addition, the experimental phase-encoded into the meta-modulator is retrieved in Fig. 4f, demonstrating a linearly increasing phase except the small jump caused by the propagation phase. Despite the existence of the propagation phase, the deviation between the retrieved and simulated phase is still smaller than 0.3*π* (Fig. 4g), which has no significant influence on convolutional results (see Fig. S3).

### The optical performance of singlet meta-imager

A singlet meta-imager (see its phase and amplitude profiles in Fig. 5b) realizing the edge-detection operator (Eq. 3) with *w*_0_ = 1.75 μm is exemplified here for the purpose of demonstration. After the discretization with five-level amplitude and 128-level phase, our fabricated singlet meta-imager has a reflective microscopic pattern with five colored rings (Fig. 3c). When illuminated by a circularly polarized laser, the singlet meta-imager has the increasing transmission (cross-polarized) from the center to the outmost ring (Fig. 3d). Figure 3e provides a quantitative comparison between the simulated and experimental amplitude profiles, where their high consistency implies an efficient amplitude modulation. In contrast, the experimental measurement of the phase is difficult because the lensing phase makes the light beam focused tightly so the interference approach is not suitable.

## Supplementary information

Supplementary Materials

Supplementary Video 1

Supplementary Video 2
