# Supplementary material for: Ultracompact meta-imagers for arbitrary all-optical convolution
Source: Light Sci Appl. 2022 Mar 18;11:62. doi: 10.1038/s41377-022-00752-5 (PMC8933501; doi:10.1038/s41377-022-00752-5)
Supplement: Supplementary file 1 — Supplementary Materials [file 41377_2022_752_MOESM1_ESM.docx]

Supplementary Materials for

**Ultracompact Meta-imagers for Arbitrary All-optical Convolution**

*Weiwei Fu^1,#^, Dong Zhao^1,#^,Ziqin Li^1,#^, Songde Liu^2,3^, Chao Tian^2,3*^, Kun Huang^1,*^*

**Affiliations:**

*^1^*Department of Optics and Optical Engineering, University of Science and Technology of China, Hefei, Anhui 230026, China

*^2^*Institute of Artificial Intelligence, Hefei Comprehensive National Science Center, Hefei, Anhui 230088, China

*^3^*Department of Precision Machinery and Precision Instrumentation, University of Science and Technology of China, Hefei, Anhui 230026, China

*^#^ W.F., D. Z., and Z. L.* contributed equally to this work.

^*^Corresponding authors: K. H. ([huangk17@ustc.edu.cn](mailto:huangk17@ustc.edu.cn)) or C. T. ([ctian@ustc.edu.cn](mailto:ctian@ustc.edu.cn))

**Table of Contents**

**1–Spread point function of a meta-imager** ………………………………………………………………P. 2

**2–Design of dielectric nano-bricks in geometric metasurfaces** ……………………………P. 6

**3–Effect of discrete phase and amplitude in the meta-modulator** ………………………P. 8

**4–Fabrication of the devices** …………………………………………………………………………………………P. 10

**5–Efficiency measurement of the fabricated metasurfaces** …………………………………P. 10

**6–Characterizing optical performance of the fabricated metalens**……………………P. 11

**7–Measuring phase modulation from the meta-modulator** …………………………………P. 13

**8–Optical experimental setup for doublet meta-imager** ………………………………………P. 14

**9–Experimentally extracted edges with different magnifications** ………………………P. 16

**10–Edge detection of a larger-size object via doublet meta-imager** …………………P. 17

**11–Edge enhancement via doublet meta-imager** ……………………………………………………P. 18

**12–Size effect for the denoising meta-modulator** ……………………………………………………P. 20

**13–Field-of-view of our meta-imager**…………………………………………………………………………P. 21

**14 -Spiral phase contrast microscopy**…………………………………………………………………………P. 23

**15-** **A brief introduction to convolutional neural network**……………………………………P. 23

**16–A comparison among the reported all-optical convolutional approaches**…P. 26

**1 | Spread point function of a meta-imager**


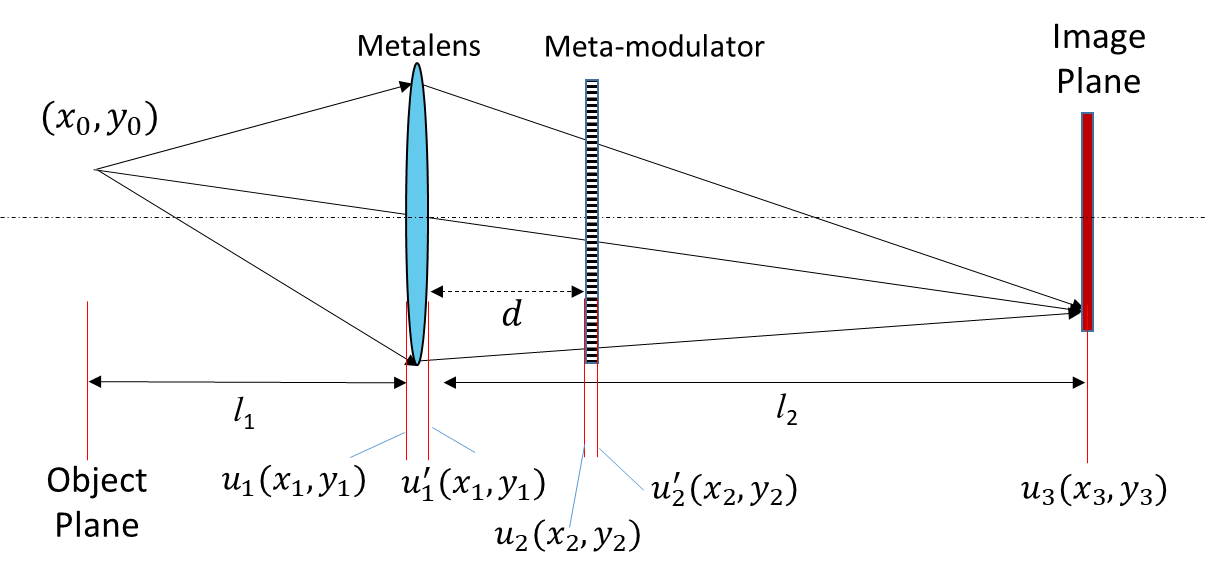


**Fig. S1.** Sketch for the pulse response of the meta-imager that containing both metalens and metamodulator.

To derive the spread point function of the meta-imager, we assume that a point source (*x*_0_,*y*_0_) located at the object plane is used to illuminate the metalens and the meta-modulator, where the spatial interval is *d*, see Fig. S1. The object and image distances are labelled by *l*_1_ and *l*_2_, respectively. Under the paraxial approximation, we have the electric field before the metalens

$u_{1}\left( x_{1}, y_{1} \right)=\frac{e^{ikl_{1}}}{l_{1}}exp\{i\frac{k}{2l_{1}}\left[ \left( x_{1}-x_{0} \right)^{2}+\left( y_{1}-y_{0} \right)^{2} \right]\}$, (S1)

where $\lambda$ is the operating wavelength, *k*=2π/*λ* is the wave number, *x*_1_ and *y*_1_ are the spatial coordinates at the metalens plane. Considering that the metalens is made in a 300nm-thick film, its thickness is ignored during our derivations. The phase profile of the metalens has the form of *t_l_*=exp[-*ik*(*x*_1_^2^+*y*_1_^2^)/(2*f*)], where *f* is the focal length of the metalens. Thus, the optical field immediately passing through the metalens is given by

$$u_{1}^{'}\left( x_{1}, y_{1} \right)=u_{1}\left( x_{1}, y_{1} \right) t_{l}\left( x_{1}, y_{1} \right)$$

$=\frac{e^{ikl_{1}}}{l_{1}}\exp\left\{ i\frac{k}{2l_{1}}\left[ \left( x_{1}-x_{0} \right)^{2}+\left( y_{1}-y_{0} \right)^{2} \right] \right\} exp[-i\frac{k}{2f}\left( {x_{1}}^{2}+{y_{1}}^{2} \right)]$

$=\frac{e^{ikl_{1}}}{l_{1}}\exp(-ik\frac{{x_{1}}^{2}+{y_{1}}^{2}}{2l_{2}} ) \exp(ik\frac{{x_{0}}^{2}+{y_{0}}^{2}}{2l_{1}} )\exp(-ik\frac{x_{1}x_{0}+{y_{1}y}_{0}}{l_{1}} ),$ (S2)

where the imaging formula $\frac{1}{l_{1}}+\frac{1}{l_{2}}=\frac{1}{f}$ is employed. Then, after propagating over a distance of *d*, the optical field $u_{2}\left( x_{2}, y_{2} \right)$ at the $x_{2}$-$y_{2}$plane (where the meta-modulator is located) can be approximated by using Fresnel diffraction

$$u_{2}\left( x_{2}, y_{2} \right)=\frac{e^{ikd}}{i\lambda d}\iint_{-\infty}^{+\infty} u_{1}^{'}\left( x_{1}, y_{1} \right) exp\{i\frac{k}{2d}\left[ \left( x_{1}-x_{2} \right)^{2}+\left( y_{1}-y_{2} \right)^{2} \right]\}dx_{1}dy_{1}$$

$$=\frac{e^{ik(l_{1}+d)}}{i\lambda l_{1}d}\exp\left( ik\frac{{x_{0}}^{2}+{y_{0}}^{2}}{2l_{1}} \right)\exp\left( ik\frac{{x_{2}}^{2}+{y_{2}}^{2}}{2d} \right)\iint_{-\infty}^{+\infty} \exp\left( -ik\frac{{x_{1}}^{2}+{y_{1}}^{2}}{2l_{2}} \right)\exp\left( ik\frac{{x_{1}}^{2}+{y_{1}}^{2}}{2d} \right)$$

$$\cdot exp \left\{ -ik\left[ x_{1}\left( \frac{x_{0}}{l_{1}}+\frac{x_{2}}{l_{2}} \right)+y_{1}\left( \frac{y_{0}}{l_{1}}+\frac{y_{2}}{l_{2}} \right) \right] \right\}dx_{1}dy_{1}$$

$=\frac{e^{ik(l_{1}+d)}}{i\lambda l_{1}d}\exp\left( ik\frac{{x_{0}}^{2}+{y_{0}}^{2}}{2l_{1}} \right)\exp\left( ik\frac{{x_{2}}^{2}+{y_{2}}^{2}}{2d} \right)\iint_{-\infty}^{+\infty} exp(ikar_{1}^{2})exp[-ik\left( x_{1}b+ y_{1}c \right)]dx_{1}dy_{1}$

$$=\frac{e^{ik(l_{1}+d)}}{i\lambda l_{1}d}\exp\left( ik\frac{{x_{0}}^{2}+{y_{0}}^{2}}{2l_{1}} \right)\exp\left( ik\frac{{x_{2}}^{2}+{y_{2}}^{2}}{2d} \right)$$

$$\cdot\int_{0}^{+\infty} \int_{0}^{2\pi} \exp\left( ikar_{1}^{2} \right)\exp\left[ -ikr_{1}\left( b\cos\varphi_{1}+c\sin\varphi_{1} \right) \right]r_{1}dr_{1}d\varphi_{1}$$

$$=\frac{e^{ik(l_{1}+d)}}{i\lambda l_{1}d}\exp\left( ik\frac{{x_{0}}^{2}+{y_{0}}^{2}}{2l_{1}} \right)\exp\left( ik\frac{{x_{2}}^{2}+{y_{2}}^{2}}{2d} \right)$$

$$\cdot\int_{0}^{+\infty} \int_{0}^{2\pi} exp(ikar_{1}^{2})exp[-ikr_{1}\sqrt{b^{2}+c^{2}}\cos\left( \varphi_{1}-{\varphi_{1}}^{'} \right)]r_{1}dr_{1}d\varphi_{1}$$

$=\frac{{ke}^{ik(l_{1}+d)}}{il_{1}d}\exp\left( ik\frac{{x_{0}}^{2}+{y_{0}}^{2}}{2l_{1}} \right)\exp\left( ik\frac{{x_{2}}^{2}+{y_{2}}^{2}}{2d} \right)\cdot\int_{0}^{+\infty} \exp\left( ikar_{1}^{2} \right)J_{0}(kr_{1}\sqrt{b^{2}+c^{2}})r_{1}dr_{1}$, (S3)

where $a=-\frac{1}{2l_{2}}+\frac{1}{2d}$, $b=\frac{x_{0}}{l_{1}}+\frac{x_{2}}{d}$, $c=\frac{y_{0}}{l_{1}}+\frac{y_{2}}{d}$, $x_{1}=r_{1}\cos\varphi_{1}$, $y_{1}=r_{1}\sin\varphi_{1}$, $cos\varphi_{1}^{'}=b/{\sqrt{b^{2}+c^{2}}}$ and $sin\varphi_{1}^{'}=c/{\sqrt{b^{2}+c^{2}}}$. In Eq. (S3), the integral over the angle coordinate *φ*_1_ is simplified by using an equality $\int_{0}^{2\pi} exp[-ixcos\left( \tau\right)]d\tau=2\pi J_{0}(x)$, where $J_{0}$is the zero-order Bessel function of the first kind. To simplify Eq. (3) further, we use another mathematical equality^1^ $\int_{0}^{+\infty} x^{v+1} e^{\pm i\alpha x^{2}}J_{v}\left( \beta x \right)dx=\frac{\beta^{v}}{\left( 2\alpha\right)^{v+1}}exp\left[ \pm i\left( \frac{v+1}{2}\pi-\frac{\beta^{2}}{4\alpha} \right) \right]$, where *α*>0, -1<Re(*v*)<1/2 and *β*>0. Thus, by using *v*=0, $\alpha=ka$ and$\beta=k\sqrt{b^{2}+c^{2}}$, Eq. (3) is expressed as

$$u_{2}\left( x_{2}, y_{2} \right)=\frac{{ke}^{ik(l_{1}+d)}}{il_{1}d}\exp\left( ik\frac{{x_{0}}^{2}+{y_{0}}^{2}}{2l_{1}} \right)\exp\left( ik\frac{{x_{2}}^{2}+{y_{2}}^{2}}{2d} \right)\frac{1}{2\alpha}exp[i(\frac{\pi}{2}-\frac{\beta^{2}}{4\alpha})]$$

$$=\frac{{ke}^{ik(l_{1}+d)}}{{2l}_{1}d}\exp\left( ik\frac{{x_{0}}^{2}+{y_{0}}^{2}}{2l_{1}} \right)\exp\left( ik\frac{{x_{2}}^{2}+{y_{2}}^{2}}{2d} \right)\frac{1}{\alpha}exp(-i\frac{\beta^{2}}{4\alpha})$$

$$=\frac{{ke}^{ik(l_{1}+d)}}{{2l}_{1}d}\exp\left( ik\frac{{x_{0}}^{2}+{y_{0}}^{2}}{2l_{1}} \right)\exp\left( ik\frac{{x_{2}}^{2}+{y_{2}}^{2}}{2d} \right)\frac{1}{k\left( -\frac{1}{2l_{2}}+\frac{1}{2d} \right)}exp[-ik\frac{\left( b^{2}+c^{2} \right)}{4\left( -\frac{1}{2l_{2}}+\frac{1}{2d} \right)}]$$

$$=\frac{{ke}^{ik(l_{1}+d)}}{{2l}_{1}d}\exp\left( ik\frac{{x_{0}}^{2}+{y_{0}}^{2}}{2l_{1}} \right)\exp\left( ik\frac{{x_{2}}^{2}+{y_{2}}^{2}}{2d} \right)\frac{1}{k\left( -\frac{1}{2l_{2}}+\frac{1}{2d} \right)}exp[-ik\frac{{(\frac{x_{0}}{l_{1}}+\frac{x_{2}}{d})}^{2}+{(\frac{y_{0}}{l_{1}}+\frac{y_{2}}{d})}^{2}}{2\left( -\frac{1}{l_{2}}+\frac{1}{d} \right)}]$$

$=e^{ik\left( l_{1}+d \right)}\exp\left( ik\frac{{x_{0}}^{2}+{y_{0}}^{2}}{2l_{1}} \right)\exp\left( ik\frac{{x_{2}}^{2}+{y_{2}}^{2}}{2d} \right)\frac{M}{l_{2}-d}$ $\cdot exp\{-ik\frac{l_{2}}{2\left( l_{2}-d \right)d}\left[ \left( x_{2}+\frac{d}{l_{1}}x_{0} \right)^{2}+\left( y_{2}+\frac{d}{l_{1}}y_{0} \right)^{2} \right]$

$$=e^{ik\left( l_{1}+d \right)}\exp\left( ik\frac{{x_{0}}^{2}+{y_{0}}^{2}}{2l_{1}} \right)\exp\left( ik\frac{{x_{2}}^{2}+{y_{2}}^{2}}{2d} \right)\frac{M}{l_{2}-d}\exp\left[ -ik\frac{\left( x_{2}+M_{1}x_{0} \right)^{2}+\left( y_{2}+M_{1}y_{0} \right)^{2}}{2M_{2}d} \right]$$

$=e^{ik\left( l_{1}+d \right)}\exp\left( ik\frac{{x_{0}}^{2}+{y_{0}}^{2}}{2l_{1}} \right)\exp\left( ik\frac{{x_{2}}^{2}+{y_{2}}^{2}}{2d} \right)\cdot\frac{M}{l_{2}-d}\cdot\exp\left\{ \frac{-ikM_{2}}{2d}\left[ \left( \frac{x_{2}+M_{1}x_{0}}{M_{2}} \right)^{2}+\left( \frac{y_{2}+M_{1}y_{0}}{M_{2}} \right)^{2} \right] \right\}$, (S4)

where we define $M_{1}=d/{l_{1}}, M_{2}={(l_{2}-d)}/{l_{2}}$and $M={l_{2}}/{l_{1}}$. According to the definition, *M*_1_ denotes the scaling factor between the spatial coordinates at the initial object and meta-modulator planes. Correspondingly, *M* is the magnification of this entire meta-imager. Note that, the aperture of the metalens is not considered in Eq. (S4) for the purpose of simplifying the calculation. In Eq. (S4), since the item (*d*-*d*^2^/*l*_2_) is smaller than *d*, the phase at the *x*_2_-*y*_2_ plane is dominated by the item $\frac{1}{k\left( -\frac{1}{2l_{2}}+\frac{1}{2d} \right)}exp[-ik\frac{{(\frac{x_{0}}{l_{1}}+\frac{x_{2}}{d})}^{2}+{(\frac{y_{0}}{l_{1}}+\frac{y_{2}}{d})}^{2}}{2\left( -\frac{1}{l_{2}}+\frac{1}{d} \right)}]$, which indicates the phase of a convergent spherical wave and therefore agrees with the predictions by geometric optics. It means that the aperture of the metalens is projected onto the meta-modulator plane (*i.e.*, *x*_2_-*y*_2_ plane) with a scaling and shifting factor, so that its equivalent aperture of a metalens at the *x*_2_-*y*_2_ plane can be approximated as $P\left( \frac{x_{2}+M_{1}x_{0}}{M_{2}},\frac{y_{2}+M_{1}y_{0}}{M_{2}} \right)$, where the scaling (*M*_2_) and shifting (*M*_1_*x*_0_and *M*_1_*y*_0_) factors are inherited from Eq. (S4).

If the meta-modulator has a complex amplitude of $h(x_{2},y_{2})$, the electric field immediately after the meta-modulator can be written as

${u_{2}}^{'}\left( x_{2}, y_{2} \right)=u_{2}\left( x_{2}, y_{2} \right)P\left( \frac{x_{2}+M_{1}x_{0}}{M_{2}},\frac{y_{2}+M_{1}y_{0}}{M_{2}} \right)h\left( x_{2},y_{2} \right)$. (S5)

Thus, by using the Fresnel diffraction, we have the electric field at the image plane

$$u_{3}\left( x_{3}, y_{3} \right)=\frac{e^{ik\left( l_{2}-d \right)}}{i\lambda{(l}_{2}-d)}\iint_{-\infty}^{+\infty} {u_{2}}^{'}\left( x_{2}, y_{2} \right)\exp\left\{ i\frac{k}{2{(l}_{2}-d)}\left[ \left( x_{3}-x_{2} \right)^{2}+\left( y_{3}-y_{2} \right)^{2} \right] \right\}dx_{2}dy_{2}$$

$=\frac{Me^{ik\left( l_{2}+l_{1} \right)}}{i\lambda{{(l}_{2}-d)}^{2}}\exp\left( ik\frac{{x_{0}}^{2}+{y_{0}}^{2}}{2l_{1}} \right)\exp[ik\frac{{x_{3}}^{2}+{y_{3}}^{2}}{2(l_{2}-d)}]\iint_{-\infty}^{+\infty} \exp\left( ik\frac{{x_{2}}^{2}+{y_{2}}^{2}}{2d} \right)exp\{\frac{-ikM_{2}}{2d}[\left( \frac{x_{2}+M_{1}x_{0}}{M_{2}} \right)^{2}+\left( \frac{y_{2}+M_{1}y_{0}}{M_{2}} \right)^{2}]\}P(\frac{x_{2}+M_{1}x_{0}}{M_{2}},\frac{y_{2}+M_{1}y_{0}}{M_{2}})h(x_{2},y_{2})\exp\left[ ik\frac{{x_{2}}^{2}+{y_{2}}^{2}}{2\left( l_{2}-d \right)} \right]exp[-ik\frac{x_{2}x_{3}+y_{2}y_{3}}{(l_{2}-d)}]dx_{2}dy_{2}$, (S6)

To simplify Eq. (S6) further, we use$x^{'}=\frac{x_{2}+M_{1}x_{0}}{M_{2}}, y^{'}=\frac{y_{2}+M_{1}y_{0}}{M_{2}}$, which yield $x_{2}=M_{2}x^{'}-M_{1}x_{0}, y_{2}=M_{2}y^{'}-M_{1}y_{0}$ , $dx_{2}=M_{2}dx^{'}$ and $dy_{2}=M_{2}dy^{'}$. Then, the electric field *u*_3_ is updated as:

$$u_{3}\left( x_{3}, y_{3} \right)=\frac{Me^{ik\left( l_{2}+l_{1} \right)}}{i\lambda{{(l}_{2}-d)}^{2}}\exp\left( ik\frac{{x_{0}}^{2}+{y_{0}}^{2}}{2l_{1}} \right)\exp\left[ ik\frac{{x_{3}}^{2}+{y_{3}}^{2}}{2\left( l_{2}-d \right)} \right]\iint_{-\infty}^{+\infty} \exp\left( ik\frac{{x_{2}}^{2}+{y_{2}}^{2}}{2M_{2}d} \right)$$

$$\exp\left[ \frac{-ikM_{2}}{2d}\left( {x^{'}}^{2}+{y^{'}}^{2} \right) \right]P\left( x^{'},y^{'} \right)h\left( x_{2},y_{2} \right)\exp\left[ -ik\frac{x_{2}x_{3}+y_{2}y_{3}}{\left( l_{2}-d \right)} \right]dx_{2}dy_{2}$$

$$=\frac{Me^{ik\left( l_{2}+l_{1} \right)}}{i\lambda{{(l}_{2}-d)}^{2}}\exp\left( ik\frac{{x_{0}}^{2}+{y_{0}}^{2}}{2l_{1}} \right)\exp\left[ ik\frac{{x_{3}}^{2}+{y_{3}}^{2}}{2\left( l_{2}-d \right)} \right]\iint_{-\infty}^{+\infty} \exp\left[ ik\frac{\left( M_{2}x^{'}-M_{1}x_{0} \right)^{2}+\left( M_{2}y^{'}-M_{1}y_{0} \right)^{2}}{2M_{2}d} \right]$$

$$\exp\left[ \frac{-ikM_{2}}{2d}\left( {x^{'}}^{2}+{y^{'}}^{2} \right) \right]P\left( x^{'},y^{'} \right)h\left( M_{2}x^{'}-M_{1}x_{0}, M_{2}y^{'}-M_{1}y_{0} \right)\exp\left[ -ik\frac{\left( M_{2}x^{'}-M_{1}x_{0} \right)x_{3}+{(M}_{2}y^{'}-M_{1}y_{0}{)y}_{3}}{\left( l_{2}-d \right)} \right] {M_{2}}^{2}dx^{'}dy^{'}={M_{2}}^{2}\frac{Me^{ik\left( l_{2}+l_{1} \right)}}{i\lambda{{(l}_{2}-d)}^{2}}\exp\left( ik\frac{{x_{0}}^{2}+{y_{0}}^{2}}{2l_{1}} \right)\exp\left[ ik\frac{{x_{3}}^{2}+{y_{3}}^{2}}{2\left( l_{2}-d \right)} \right]\exp[ik\frac{{M_{1}}^{2}{{(x}_{0}}^{2}+{y_{0}}^{2})}{2dM_{2}}]\exp[ik\frac{M_{1}{(x}_{0}x_{3}+x_{0}y_{3})}{l_{2}-d}]$$

$$\iint_{-\infty}^{+\infty} P\left( x^{'},y^{'} \right)h\left( M_{2}x^{'}-M_{1}x_{0}, M_{2}y^{'}-M_{1}y_{0} \right)exp\{-\frac{ik}{l_{2}}[x^{'}\left( x_{3}+Mx_{0} \right)+y^{'}\left( y_{3}+My_{0} \right)]\}dx^{'}dy^{'}$$

$$={M_{2}}^{2}\frac{Me^{ik\left( l_{2}+l_{1} \right)}}{i\lambda{{(l}_{2}-d)}^{2}}\exp\left( ik\frac{{x_{0}}^{2}+{y_{0}}^{2}}{2l_{1}} \right)\exp\left[ ik\frac{{x_{3}}^{2}+{y_{3}}^{2}}{2\left( l_{2}-d \right)} \right]\exp[ik\frac{{M_{1}}^{2}{{(x}_{0}}^{2}+{y_{0}}^{2})}{2dM_{2}}]\exp[ik\frac{M_{1}{(x}_{0}x_{3}+x_{0}y_{3})}{l_{2}-d}]$$

·$\iint_{-\infty}^{+\infty} P\left( x^{'},y^{'} \right)exp\{-\frac{ik}{l_{2}}[x^{'}\left( x_{3}+Mx_{0} \right)+y^{'}\left( y_{3}+My_{0} \right)]\}dx^{'}dy^{'}$

$$\otimes\iint_{-\infty}^{+\infty} h\left( M_{2}x^{'}-M_{1}x_{0}, M_{2}y^{'}-M_{1}y_{0} \right)exp\{-\frac{ik}{l_{2}}[x^{'}\left( x_{3}+Mx_{0} \right)+y^{'}\left( y_{3}+My_{0} \right)]\}dx^{'}dy^{'}$$

$${{=M}_{2}}^{2}\frac{Me^{ik\left( l_{2}+l_{1} \right)}}{i\lambda{{(l}_{2}-d)}^{2}}\exp\left( ik\frac{{x_{0}}^{2}+{y_{0}}^{2}}{2l_{1}} \right)\exp\left[ ik\frac{{x_{3}}^{2}+{y_{3}}^{2}}{2\left( l_{2}-d \right)} \right]\exp[ik\frac{{M_{1}}^{2}{{(x}_{0}}^{2}+{y_{0}}^{2})}{2dM_{2}}]\exp[ik\frac{M_{1}{(x}_{0}x_{3}+x_{0}y_{3})}{l_{2}-d}]$$

$\cdot\tilde{P}\otimes\tilde{h}$, (S7)

where

$\tilde{P}\mathcal{=F}\left[ P\left( x^{'},y^{'} \right) \right]=\tilde{P}\left( f_{x}, f_{y} \right)|_{f_{x}=\frac{x_{3}+Mx_{0}}{\lambda l_{2}}, f_{y}=\frac{y_{3}+My_{0}}{\lambda l_{2}}}$ , (S8)

$$\tilde{h}=\iint_{-\infty}^{+\infty} h\left( M_{2}x^{'}-M_{1}x_{0}, M_{2}y^{'}-M_{1}y_{0} \right)exp\{-\frac{ik}{l_{2}}[x^{'}\left( x_{3}+Mx_{0} \right)+y^{'}\left( y_{3}+My_{0} \right)]\}dx^{'}dy^{'}$$

$$=\iint_{-\infty}^{+\infty} h\left( x_{2}, y_{2} \right)exp\{-\frac{ik}{l_{2}}\left[ \frac{x_{2}+M_{1}x_{0}}{M_{2}}\left( x_{3}+Mx_{0} \right)+\frac{y_{2}+M_{1}y_{0}}{M_{2}}\left( y_{3}+My_{0} \right) \right]\}\frac{1}{{M_{2}}^{2}}dx_{2}dy_{2}$$

$$=\frac{1}{{M_{2}}^{2}}exp[-ik\frac{M_{1}x_{0}\left( x_{3}+Mx_{0} \right)+M_{1}y_{0}\left( y_{3}+My_{0} \right)}{l_{2}M_{2}}]\iint_{-\infty}^{+\infty} h\left( x_{2}, y_{2} \right)$$

$$\cdot exp[-ik\frac{x_{2}\left( x_{3}+Mx_{0} \right)+y_{2}\left( y_{3}+My_{0} \right)}{l_{2}-d}]dx_{2}dy_{2}$$

$=\frac{1}{{M_{2}}^{2}}exp \left[ -ik\frac{M_{1}\left( x_{0}x_{3}+y_{0}y_{3} \right)}{l_{2}-d} \right]exp \left[ -ik\frac{{MM}_{1}\left( {x_{0}}^{2}+{y_{0}}^{2} \right)}{l_{2}M_{2}} \right]{\mathcal{F}\left[ h\left( x_{2}, y_{2} \right) \right]}_{f_{x}=\frac{x_{3}+Mx_{0}}{\lambda{(l}_{2}-d)}, f_{y}=\frac{y_{3}+My_{0}}{\lambda{(l}_{2}-d)}}$, (S9)

After substituting Eqs. (S8) and (S9) into Eq. (S7), we have

$$u_{3}\left( x_{3}, y_{3} \right)\propto\exp\left[ ik\left( \frac{1}{2l_{1}}+\frac{{M_{1}}^{2}}{2{dM}_{2}}-\frac{MM_{1}}{2l_{2}M} \right)\left( {x_{0}}^{2}+{y_{0}}^{2} \right) \right]\exp\left[ ik\frac{{x_{3}}^{2}+{y_{3}}^{2}}{2\left( l_{2}-d \right)} \right]$$

$\cdot\tilde{P}\otimes\left\{ {\mathcal{F}\left[ h\left( x_{2}, y_{2} \right) \right]}_{f_{x}=\frac{x_{3}+Mx_{0}}{\lambda{(l}_{2}-d)}, f_{y}=\frac{y_{3}+My_{0}}{\lambda{(l}_{2}-d)}} \right\}$

=$\exp\left[ ik\frac{1}{2l_{1}}\frac{l_{2}\left( f-d \right)}{f\left( l_{2}-d \right)}\left( {x_{0}}^{2}+{y_{0}}^{2} \right) \right]\exp\left[ ik\frac{{x_{3}}^{2}+{y_{3}}^{2}}{2\left( l_{2}-d \right)} \right] \cdot\tilde{P}\otimes\left\{ {\mathcal{F}\left[ h\left( x_{2}, y_{2} \right) \right]}_{f_{x}=\frac{x_{3}+Mx_{0}}{\lambda{(l}_{2}-d)}, f_{y}=\frac{y_{3}+My_{0}}{\lambda{(l}_{2}-d)}} \right\}$ , (S10)

which is the main result in this work and can be taken as the point spread function of our proposed meta-imager. Eq. (S10) indicates that the point spread function can be taken as a convolution between both Fourier transforms of the aperture function *P* and the meta-modulator *h*. Note that, the spatial frequencies in both Fourier transforms have a scaling factor of *M*_2_, which will not influence the imaging results. In addition, the phase item $\exp\left[ ik\frac{1}{2l_{1}}\frac{l_{2}\left( f-d \right)}{f\left( l_{2}-d \right)}\left( {x_{0}}^{2}+{y_{0}}^{2} \right) \right]$ leads to optical aberration, which determines the field of view. Considering that the parameter *d* has the value ranging from 0 to *l*_2_, we can remove the phase item if *d*=*f*, where our meta-imager behaves similarly with the Fourier spatial filtering. But, when *d*≠*f*, our meta-imager could also work well, which is the fundamental difference from the Fourier filtering approach. Moreover, the variable *d* enables us to decrease the volume of the entire mea-imager by using small *d*. For example, when *d*=0, both metalens and meta-modulator in the meta-imager can be integrated into a single device with complex-amplitude modulation, thus allowing for the more compact volume. The field of view for different *d* will be discussed later, see the following singlet meta-image section.

**2 | Design of dielectric nano-bricks in geometric metasurfaces**

To realize the expected phase and amplitude modulation in our meta-imager, we use dielectric geometric metasurfaces (see the unit cell sketched in Fig. S2a) ^2,3^ composed of a rotating silicon nanobrick sitting on a sapphire substrate. The nanobrick has a rotation angle of $\theta$ between its long axis and *x* axis. We can obtain its Jones matrix:

$\boldsymbol{T}\left( \theta\right)=\boldsymbol{R}\left( -\theta\right)\boldsymbol{T}_{0}\boldsymbol{R}\left( \theta\right)=\left[ \begin{matrix} \cos\theta& -\sin\theta\\ \sin\theta& \cos\theta\end{matrix} \right]\left[ \begin{matrix} t_{x} & 0 \\ 0 & t_{y} \end{matrix} \right]\left[ \begin{matrix} \cos\theta& \sin\theta\\ -\sin\theta& \cos\theta\end{matrix} \right],$ S(11)

where $\boldsymbol{R}\left( \theta\right)$ is the rotation operator, $\boldsymbol{T}_{0}$ is the Jones matrix when the nanobrick is not rotated (*i.e.*,$\theta$=0), *t_x_*=|*t_x_*|exp(*iφ_x_*) and *t_y_*=|*t_y_*|exp(*iφ_y_*) are the transmission of the *E_x_* and *E_y_* components of the incident light, respectively. If the incident light with circular polarization has the electric field $\boldsymbol{E}^{\sigma}=E\cdot(\boldsymbol{e}_{\boldsymbol{x}}+\sigma i\boldsymbol{e}_{\boldsymbol{y}}) (\sigma=\pm1$), its transmitted electric field can be expressed as:

$$\boldsymbol{E}_{trans}=\boldsymbol{T}\left( \theta\right)\boldsymbol{E}^{\sigma}=E\left[ \begin{matrix} \cos\theta& -\sin\theta\\ \sin\theta& \cos\theta\end{matrix} \right]\left[ \begin{matrix} t_{x} & 0 \\ 0 & t_{y} \end{matrix} \right]\left[ \begin{matrix} \cos\theta& \sin\theta\\ -\sin\theta& \cos\theta\end{matrix} \right]\left[ \begin{matrix} 1 \\ \sigma i \end{matrix} \right]$$

$=\frac{t_{x}+t_{y}}{2}\boldsymbol{E}^{\sigma}+\frac{t_{x}-t_{y}}{2}e^{2i\sigma\theta}\boldsymbol{E}^{-\sigma}=\left| \frac{t_{x}+t_{y}}{2} \right|\cdot e^{i\varphi_{prop}^{\sigma}}\boldsymbol{E}^{\sigma}+|\frac{t_{x}-t_{y}}{2}|\cdot e^{i\varphi_{prop}^{-\sigma}}\cdot e^{2i\sigma\theta}\boldsymbol{E}^{-\sigma}$, S(12)

where $\varphi_{prop}^{\sigma}$ and $\varphi_{prop}^{-\sigma}$ are the propagation phase of the co- and cross-polarized transmitted light, respectively, and $\tan\left( \varphi_{prop}^{\pm\sigma} \right)=(\left| t_{x} \right|sin\varphi_{x}\pm\left| t_{y} \right|sin\varphi_{y})/(\left| t_{x} \right|cos\varphi_{x}\pm\left| t_{y} \right|cos\varphi_{y})$. In Eq. S(12), although the co-polarized (*i.e.*, $\boldsymbol{E}^{\sigma}$) transmitted light has phase and amplitude modulation, both of them depend on the dimension of the nanorbricks simultaneously so that it is difficult to realize the independent phase and amplitude modulation (required mandatorily in our meta-modulator). In contrast, the cross-polarized transmitted light has the amplitude modulation of |$\frac{t_{x}-t_{y}}{2}$| and the phase modulation of $\varphi_{prop}^{-\sigma}+2\sigma\theta$, where the geometric phase $2\sigma\theta$ is independent on the geometry of the nanobrick. Therefore, in this work, we employ the geometry of nanobrick to control the amplitude while the rotation of the nanobrick for the phase modulation. Although the propagation phase $\varphi_{prop}^{-\sigma}$ also provides the additional modulation, its value has no significant variation at the chosen dimension of the nanobricks. Moreover, the contribution from the propagation phase can be compensated by using the geometric phase with an additional shift.

To simulate the conversion efficiency, we employ finite-difference time-domain (FDTD) method to calculate the electromagnetic responses. In our simulation, we use the periodic boundaries along *x* and *y* directions, while the perfect matching layers are adopted along z directions. Considering the operating wavelength of 633 nm, we use the period of 250 nm along *x* and *y* directions, thus satisfying the subwavelength pixel size required in the metasufaces. The nanobricks are made in a single-crystal silicon film (University Wafer Inc.) with a thickness of 300 nm. Its refractive index is measured experimentally by using an ellipsometer, which yields the real and imaginary data as shown in Fig. S2b.


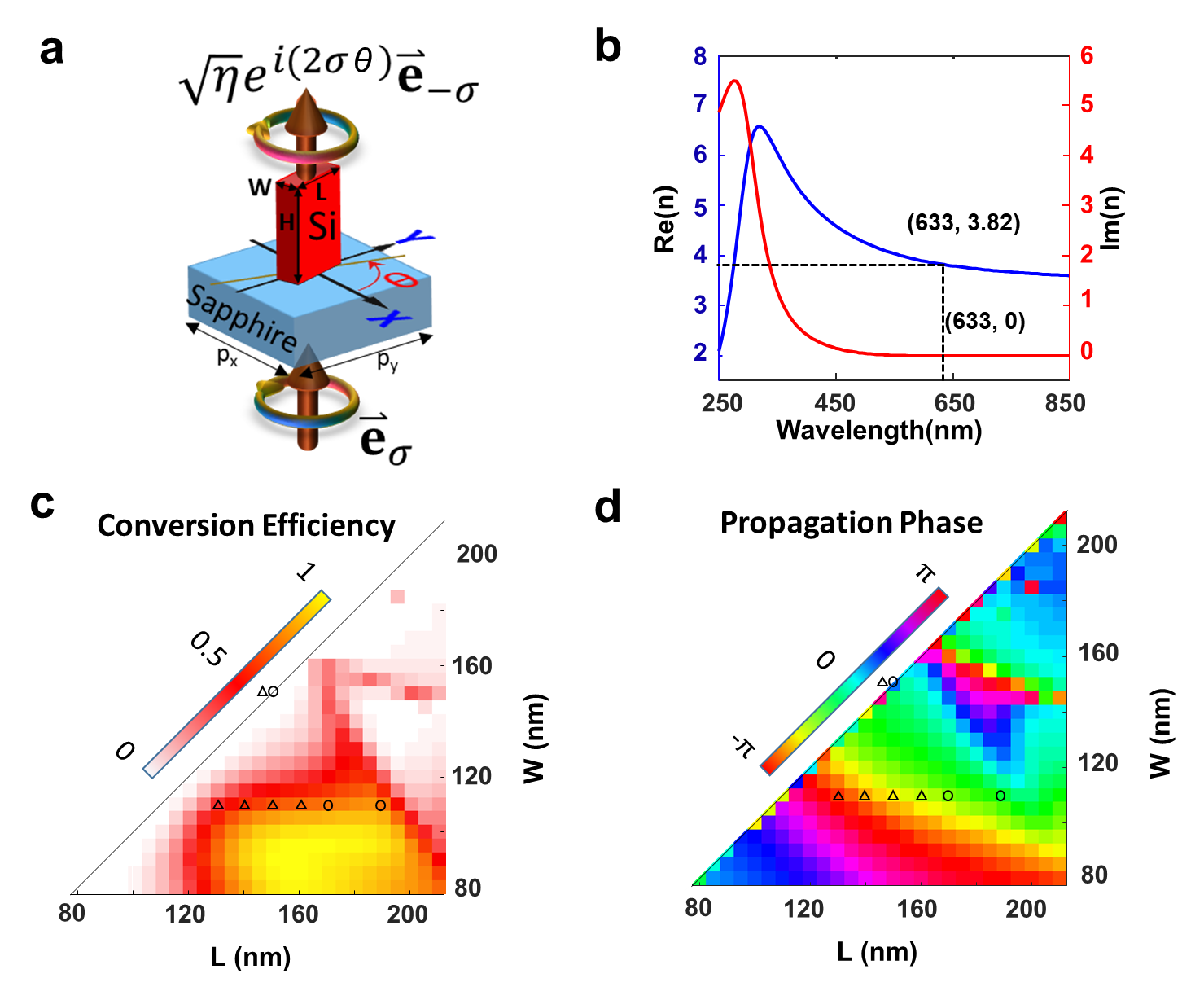


**Fig. S2 | Design of dielectric geometric metasurfaces**. **(a)** Sketch of a unit cell in dieletric geometric metasurfaces. The polarization conversion efficiency is labelled by *η*. The handedness of the circularly polarized light is labelled by *σ*, the sign of which stands for its spin. The spin of a circularly polarized light has a binary value of ±1, so that two circularly polarized light beams with different spins are orthogonal. **(b)** Real (blue) and imaginary (red) parts of experimental refractive index that is created by an ellipsometer by fitting the reflectance of the film at the broadband wavelengths. The ellipsometer (SOPRA,GES5E) offers the spectral resolution of 0.5 nm at the wavelength of 633nm under the high-resolution mode. **(c-d)** Simulated polarization efficiency **(c)** and propagation phase **(d)** of light passing through the nanobricks with different lengths (L) and widths (W). The height of the nanobrick is 300 nm, which is determined by the thickness of the silicon film. The hollow circles label the parameters of the selected nanobricks in the doublet meta-imager, while the hollow triangles for the case of the singlet meta-imager.

The simulated conversion efficiency is shown in Fig. S2c, which presents the efficiency with a large range from 0 to 1. It implies that the amplitude modulation is sufficient for our meta-imager. In this work, we realize the amplitude modulation by fixing the width of *W*=110nm, which is chosen for an easy fabrication. For *W*=110nm, the conversion efficiency has a peak around *L*=160nm, so that both increasing and falling edges can be used for amplitude modulation. But, it leads to high sensitivity to the geometry of nanobricks, hereby increasing the difficulty in fabrication. So, in this work, the discrete amplitude is used for easy fabrication. The discrete amplitude in the case of doublet meta-imager is three-level, which is realized by using the nanobricks with the dimension (*W*=110nm, *L*=170nm), (*W*=110nm, *L*=190nm) and (*W*=150nm, *L*=150nm). For the case of singlet meta-imager, we utilize five-level amplitude by using the nanorbricks with the dimension of (*W*=160 nm, *L*=110 nm), (*W*=150 nm, *L*=110 nm), (*W*=140 nm, *L*=110 nm), (*W*=130 nm, *L*=110 nm) and (*W*=150 nm, *L*=150 nm). Note that, the nanobricks with *W*=150nm, *L*=150nm are used to realize the zero-amplitude due to large dimension.

In addition, we have to emphasize that the nanobricks with different dimensions yield the propagation phase of the cross-polarized transmission light, as plotted in Fig. S2d. From the pseudo-color, we can observe the nearly identical propagation phase at the interested region of *W*=110nm, which thus avoids the significant influence to optical performance of the entire meta-imager. In fact, we also give a detailed comparison between all-optical convolutions for both cases of the continuous and discrete amplitude modulation, as discussed in the following Section 3. Importantly, the propagation phase can be removed completely in theory by adding an additional sign-opposite value into the geometric phase in each ring of discretized meta-modulator. Thus, the undesired propagation phase can be compensated by the geometric phase, so that the error from the propagation phase can be ignored.

**3 | Effect of discrete phase and amplitude in the meta-modulator**

In order to evaluate the influence from discrete amplitude and phase in the meta-modulator, we have simulated optical performance of these discretized meta-modulators by using Rayleigh-Sommerfeld diffraction, which is implemented on the basis of fast Fourier transform (see the details in our previous work^4^). The simulated results are shown in Fig. S3, where the double meta-imager with the parameters *M*=1, *d*=*f*=2.5 mm, *w*_0_ =1.5 μm and *λ*=633 nm are used in our simulations. Firstly, we test the meta-modulator for detecting the edge of an amplitude object “USTC” (see Fig. S3a). Fig. S3b presents the extracted edges when the amplitude of the meta-modulator is continuous without any discretization. In comparison, the discrete-amplitude meta-modulator also works well, which can be observed in Fig. S3c. The slight inhomogeneity existing at the edges does not have the significant influence on the quality of the convolution. In addition, to mimic the realistic case, the propagation phase has also been taken account into our simulation. The value of the employed propagation phase is obtained from Fig. S2d by addressing the dimension of the nanobricks. The good agreement between two cases of the continuous- and discrete-amplitude meta-modulator implies that the discrete amplitude has little influence on the convolutions. Similarly, the convolutions for spatial differentiation (Figs. S3d-S3f) and denoising (Figs. S3g-S3i) also show high consistence. All these simulations confirm that the discretized meta-modulators are valid to demonstrate the convolutional operations, thus confirming the feasibility of our proposed meta-imager.


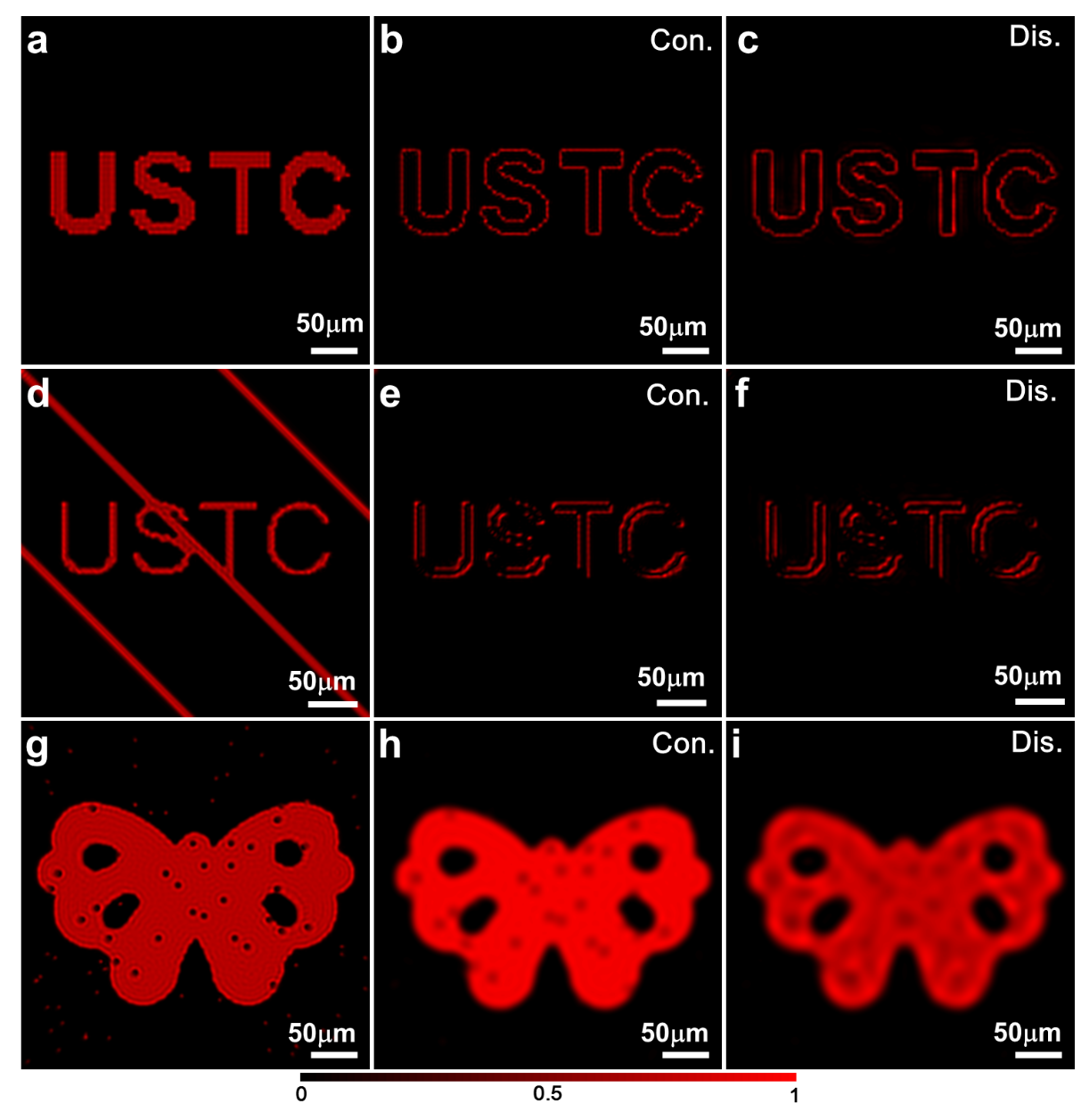


**Fig. S3 | Simulated convolutional results by using continuous and discrete meta-modulator. (a-c)** Outputted edge detection for different cases: without meta-modulator **(a)**, with continuous **(b)** and discrete **(c)** meta-modulator. The image without the meta-modulator is shown here as a control case that presents the validity of convolutional operation. The parameters are *M*=1, *d*=*f* (*f*=2.5mm) and *w*_0_ =1.5 μm at 633 nm. The detection accuracy *w*_0_ of the convolution operator encoded in the meta-modulator is sketched in Fig. 2(a) of main text. Scalebars: 50 μm. **(d-f)** One-dimensional spatial differentiation for different cases: without meta-modulator **(d);** with continuous **(e)** and discrete **(f)** meta-modulator. Here, the used parameters are *M*=1, *d*=*f* (*f*=2.5mm) and *w*_0_ =1.5 μm at 633 nm. Scalebars: 50 μm. **(g-i)** Denoising operation for different cases: without meta-modulator **(g)**; with continuous **(h)** and discrete **(i)** meta-modulator. The relative parameters are *M*=1, *d*=*f* (*f*=2.5mm) and *w*_0_ =4 μm at 633 nm. Scalebars: 50 μm. All three meta-modulators in doublet meta-imagers throughout this work have discretized amplitude with three levels: 1, 0.5 and 0 and phase with 128-level phase. In the simulations, the propagation phase is taken account in discrete meta-modulator, where the maximum propagation phase is smaller than 0.3π.

**4 | Fabrication of the devices**

In this work, the designed metalenses and meta-modulators are fabricated through standard nano-fabrication process. The sapphire substrate is first deposited with a 300 nm-thick single-crystal silicon film, which determines the height of the designed Si nanorods. Then, the positive electron-beam resist with the thickness of 100 nm is coated and baked on the silicon film. Subsequently, the photoresist is patterned with the mask carrying the structural information of meta-devices by using electron beam lithography (JEOL, JBX 6300FS) with an accelerating voltage of 100 kV. The patterned photoresist is developed, followed by the deposition of a 15 nm-thick chromium as hard mask. Thus, the nanobrick patterns are transferred into the ultrathin chromium film after lift-off. Next, the 300 nm-thick Si layer was etched by an Inductively Coupled Plasma etcher (Oxford, Plasma Pro System100 ICP380). Finally, the residual chromium mask is removed using dry etching technique, yielding the expected metasurfaces.

**5 | Efficiency measurement of the fabricated metasurfaces**


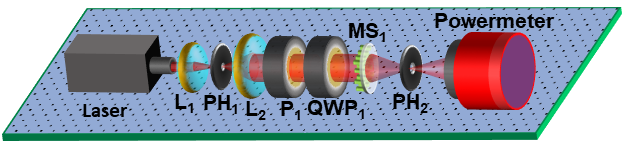


**Fig. S4 |** **Sketch of the experimental setup for measuring the efficiency of the metalens**. Laser: wavelength 633 nm; L1: lens with focal length 35 mm; L2: lens with focal length 75 mm; P: polarizer. QWP: quarter-wave plate; PH: pinhole; MS1: metalens;

In order to measure the efficiency of the fabricated metalens (contained in the doublet meta-imager), we have made the experimental setup, as sketched in Fig. S4. A laser beam with the wavelength of 633 nm is reshaped by using a telescope system composed of two lenses (L_1_ and L_2_) and a pinhole (PH_1_), yielding a fundamental Gaussian beam with its lateral size of ~1mm in diameter. To create the required circularly polarized light, a linear polarizer (P_1_) and a quarter waveplate are employed together to tune the polarization of the Gaussian beam precisely as the incidence of the metalens. Due to the strong birefringence in the sapphire substrate, the incident light is used to illuminate the nanobricks side of the sample firstly (see Fig. S4) before going through the sapphire substrate. Due to the large focal length of the metalens, the focal spot is located outside the substrate so that a pinhole (PH_2_) with the size of 50 μm is placed on the focal plane to filter out the focused light, which is the converted light with crossed polarization and then recorded as I_1_ by a power meter (Fig. S4). When both the metalens and the pinhole (PH_2_) are removed so that the Gaussian beam is incident on the bare sapphire substrate, the transmitted light through the sapphire is taken as the incident power I_0_ and recorded by the powermeter. Thus, the experimental focusing efficiency of the metalens is evaluated by using *η*=I_1_/I_0_, yielding the efficiency of 71.4%.

In addition, the experimental efficiency of the metasurfaces has also been characterized by fabricating a series of nanobrick arrays (where each array has the size of 100 μm×100 μm) with dimension-different nanobricks. Due to the small size of nanobrick array, its efficiency measurement is implemented with the help of the objective lens (for image magnification) and CCD (for recording intensity), where the detailed steps during the measurement is completely the same with those in our previous work and therefore not discussed here. The measured efficiency is used to plot Figure 1c in the main text.

**6 | Characterizing optical performance of the fabricated metalens**

To verify the focusing performance of the metalens, we firstly measure the intensity distribution near the focal plane by recording the intensity profiles with a CCD camera after the magnified by an objective lens. The measured intensity along the cross-section (*x-z*) plane is shown in Fig. S5a, which has a good agreement with our simulations in Fig. S5b. Note that, the achieved focal plane is located at the z=~2465 μm, which has a deviation of 35μm from the expected focal length of *f*=2500μm. It originates from the paraxial approximation of the phase profiles ($exp[-{ikr^{2}}/{2f}]$) encoded in the meta-lens. During the simulation of the intensity profiles, the rigorous Rayleigh-Sommerfeld diffraction integral is employed without the approximation, hereby creating the above discrepancy. Nevertheless, it has no significant influence on the imaging performance, which can be observed by its imaging result of a binary-amplitude object (“rabbit” etched through an opaque chromium film) in Fig. S5c. The image with a magnification of 1 is projected by the metalens onto the CCD camera. The good homogeneity and high contrast can be obtained without any distortion, indicating good imaging quality.

**
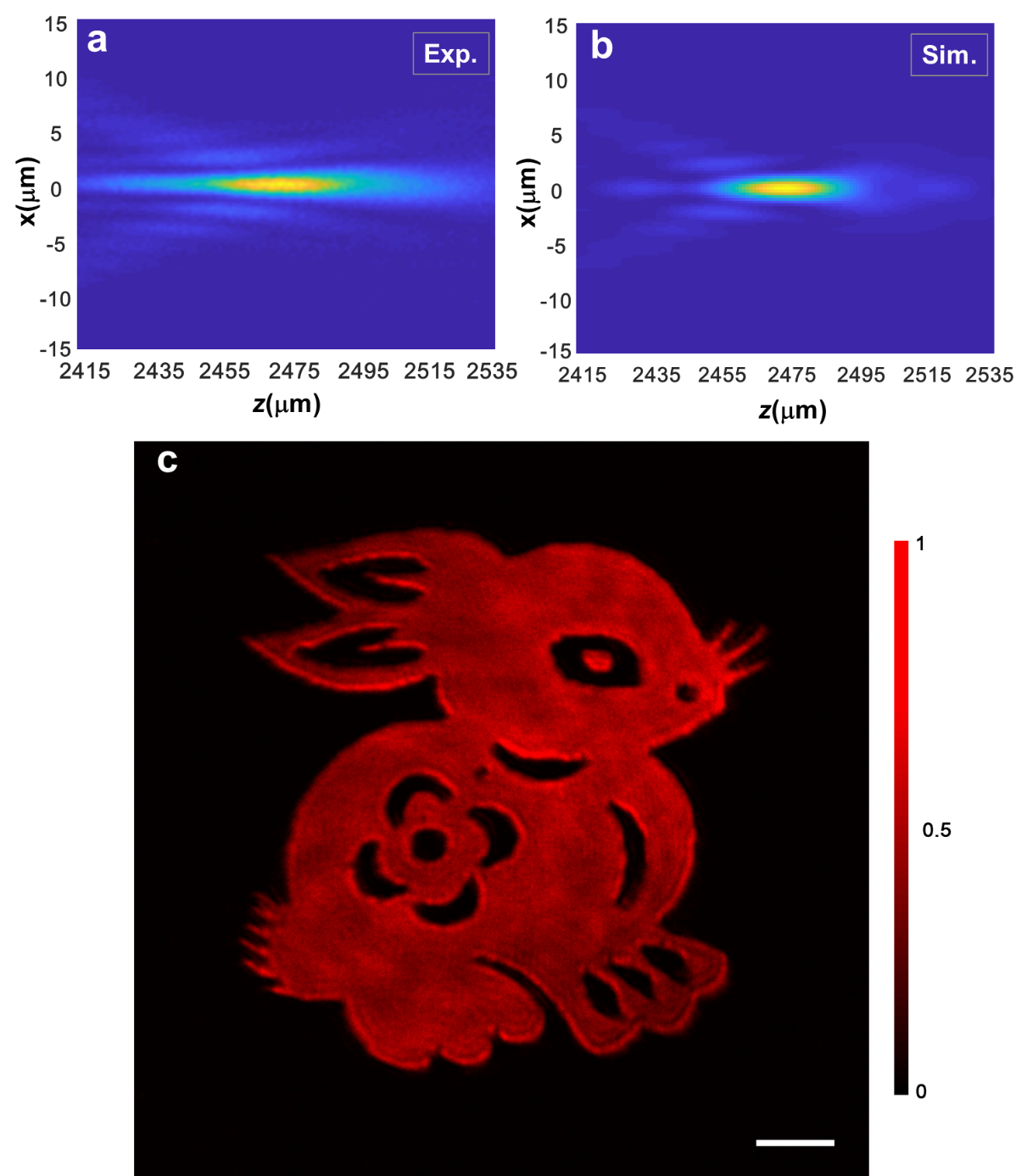
**

**Fig. S5 | Characterizing the focusing and imaging performance of the metalens. (a-b)** Energy intensity distribution near the focal plane of the metalens at different propagation distances *z* from 2415 μm to 2535 μm in experiment **(a)** and simulation **(b)**. **(c)** Measured images by using a single metalens. Scalebar: 50 μm.

**7 | Measuring phase modulation from the meta-modulator**

**
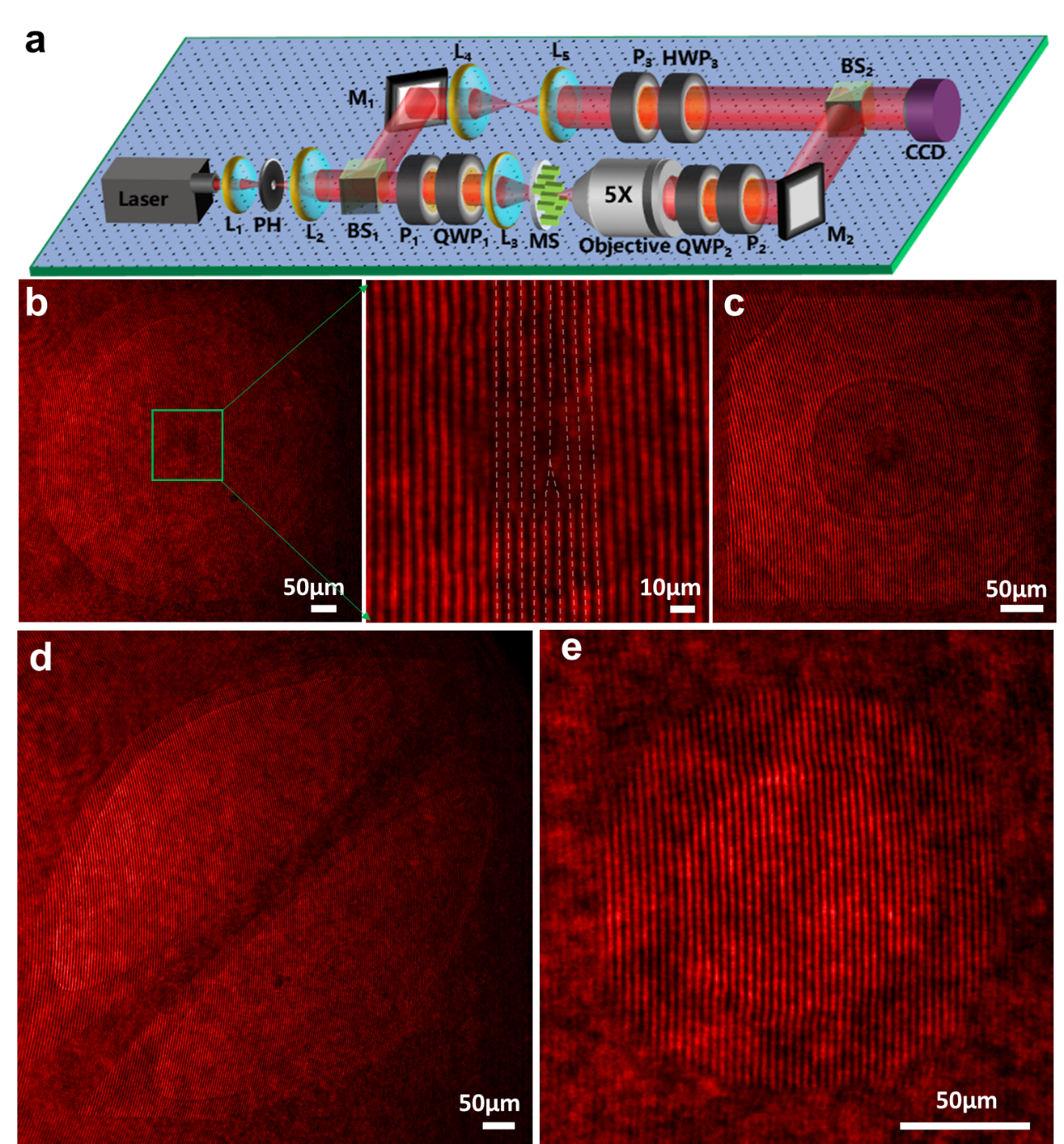
**

**Fig. S6 | Phase measurement. (a)** Sketch of the experimental setup for characterizing the phase in a self-built Mach-Zehnder interferometer**.** Amplified signal beam after passing metasurfaces interferes with the equal-intensity references beam for obtaining high-contrast interference patterns. Laser: wavelength 633 nm. L_1_: lens with a focal length of 35 mm; L_2_: lens with a focal length of 75 mm. PH: pinhole; P: polarizer; QWP: quarter-wave plate; HWP: Half-wave plate; BS: beam splitter (50:50). M: mirror. L_3_: lens with a focal length of 100 mm; L_4_: lens with a focal length of 75 mm; L_5_: lens with a focal length of 100mm; MS: metasurfaces; CCD: Charge Coupled Device. **(b-e)** Measured interference patterns for different meta-modulators that realize edge detection **(b)**, edge enhancement **(c)**, spatial differentiation **(d)** and denoising **(e)**.

To measure the phase encoded in the meta-modulators, a modified Mach-Zehnder interferometer (See Fig. S6a is used here to check the proformance of meta-modulators. A high-coherence laser with the wavelength 633 nm is expanded by a telescopic system compose of two lenses (L_1_ and L_2_), where a pinhole (PH) of 15 μm in diameter is located at their confocal plane for obtaining a quasi-Gaussian beam that is then divided into the signal and reference beam via a beam splitter (BS_1_). In the signal beam, the polarizer (P_1_) and quarter-wave plate (QWP_1_) are used to produce the required circularly polarized light, which is then focused weakly by a lens (L_3_) onto the entire meta-structures, so as to increase the transmitted power through the metasurfaces. Then, a 5× objective lens is used to project the image of the meta-modulator onto the CCD camera after going through a circular-polarization analyzer (composed of a quarter-waveplate (QWP_2_) and linear polarizer (P_2_) to remove the co-polarized part of the transmitted light) and being guided by a mirror (M_2_) and a beam splitter (BS_2_). On the other hand, the reference beam is expanded further by another telescopic system (composed of two lenses L_4_ and L_5_) to match the dimension of the magnified meta-modulator for a better interference. Then, an additional polarizer (P_3_) is used to control the intensity of the reference beam because its incident light is linearly polarized. The following half-wave plate (HWP_3_) can rotate the direction of the linearly polarized light, so as to keep the identical polarization between the reference and signal beams for high-visibility interference fringes. Finally, the signal beam and the reference beam are combined together by a beam splitter (BS_2_, 50:50), where the resulting interference are recorded by a color camera. The captured interference fringes are shown in Figs. S6b-S6e by addressing their functionalities such as edge detection, edge enhancement, spatial differentiation and pepper-salt denoising.

To retrieve the phase from these interference pattern, we use a fast Fourier transform (FFT) that could separate the spectrum (±1 orders) of the signal beam from that (0 order) of the reference beam. Next, only the spectrum of the signal beam is maintained and shifted to the center of the spectrum domain, thus creating a new spectrum whose inverse FFT gives the retrieved phase. The detailed information about the phase retrieval can be found in our previous works^5,6^.

**8 | Optical experimental setup for doublet meta-imager**

To characterize the convolutional operation, we employ the experimental setup in Fig. S7a. A laser beam with the wavelength of 633 nm goes through the beam-shaping system and the circular polarizer (composed of a linear polarizer P_1_ and a quarter waveplate QWP_1_) successively, creating the required circular polarized beam that operates as the incidence of the object. The position of the object determines the imaging magnification, which can be tuned in experiment. Light passing through the object is collected by the metalens and modulated by the meta-modulator, forming the convolutional results at the image plane. To record it, an objective lens with magnification of 20 is used to project the convolutional image onto the CCD. Before recording, a cross circularly polarizer (P_2_ and QWP_2_) is used to eliminate the background light that has the same handedness as the input beam. Note that, since the meta-imager has the limited resolving power, the specimens with small details (such as the biological DNA, onion and oral epidetmal cells used in this work) are magnified by another objective lens firstly so that the corresponding magnified objects are located at the object plane of the meta-imager, for a better processing of the objects.


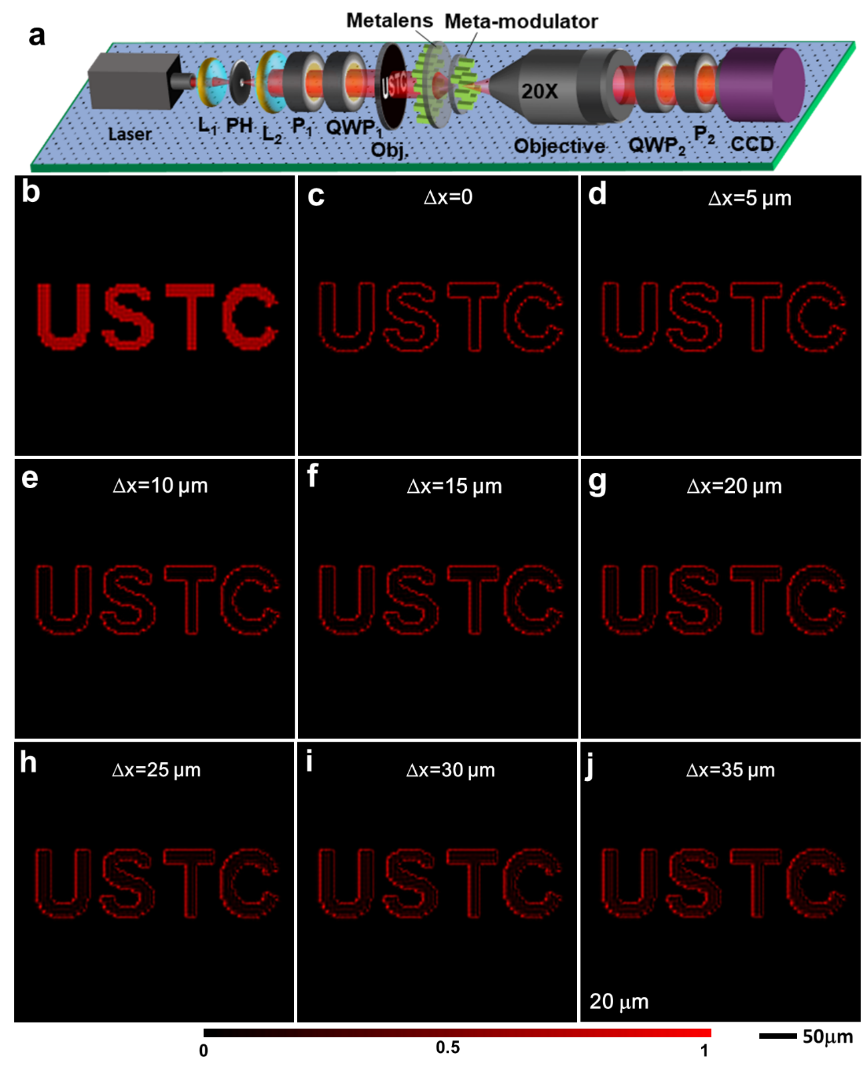


**Fig. S7 | Experimental characterization of convolutional operation via a doublet meta-imager.** **(a)** Experimental setup. The object is placed at the object plane and illuminated by a laser with wavelength 633 nm. A linear polarizer (P1) and a quarter-wave plate (QWP1) are used to fully change the incident laser beam from linear polarization to circular polarization. A 20× microscope objective is used for image magnification after passing the MS1 (metalens) and MS2 (meta-modulator) successively. A cross circularly polarizer (P2 and QWP2) is used to eliminate the background light that has the same handedness as the input beam. Laser: wavelength 633 nm. L1: lens with focal length 35 mm; L2: lens with focal length 75 mm. PH: pinhole; CCD: Charge Coupled Device. **(b)** Simulated output intensity images (with the magnification *M*=1) by the metalens without meta-modulator. **(c-j)** Simulated output intensity images using the meta-imager with parameter *w*_0_ =1.5 μm and *M*=1 when meta-modulator has different offsets (*∆x*) along the *x* direction.

In the configuration of doublet meta-imager, the alignment between metalens and meta-modualtor is important to guarantee the quality of the convolution operations. To evaluate it quantitatively, we simulate the convolutional results for edge detection for different lateral deviations ∆x between metalens and meta-modulators, by using the Rayleigh-Sommerfeld diffraction with the focal length of 1mm and magnification of 1. Fig. S7b shows the image of a binary-amplitude object “USTC”. Its detected edges are shown Figs. S7c-S7j by addressing different deviation ∆x. The simulated results indicates that the edge of the object can be extracted clearly even when ∆x=20μm, which implies that our doublet meta-imager has a tolerance to the lateral misalignment of 20μm (which can be tuned precisely by using a high-precision 3-dimensional stage). The only influence on the image quality is that the intensity contrast between the left and right edges increases with the increment of the lateral deviation ∆x, and clearer illustrations can be seen in Figs. S7h-S7j. In addition, such a lateral tolerance of 20 μm is also easily obtained if the metalens and meta-modulator can be fabricated at the front and back sides of the substrate, respectively.

**9 | Experimentally extracted edges with different magnifications**

To observe the edges with different magnifications, we tune the distance between the object and the meta-imager in experiment. The image distance is also changed so that the following imaging system is moved correspondingly for capturing the magnified or shrunken edges. Figure S8a shows the experimentally measured binary-amplitude object that is imaged with the magnification of 1 by using the metalens (*f*=2.5mm), working as a control experiment. When this object is processed by using our meta-imager, the detected edges with different magnifications from M=2.5 to M=0.5 with an interval of 0.5 are shown in Figs. S8b-S8f, respectively. In despite of the existence of the inhomogeneous intensity caused by the inevitable defects in both metalens and meta-modulator, these clear edges confirm the feasibility of the scalable convolutional operations by using our meta-imager.


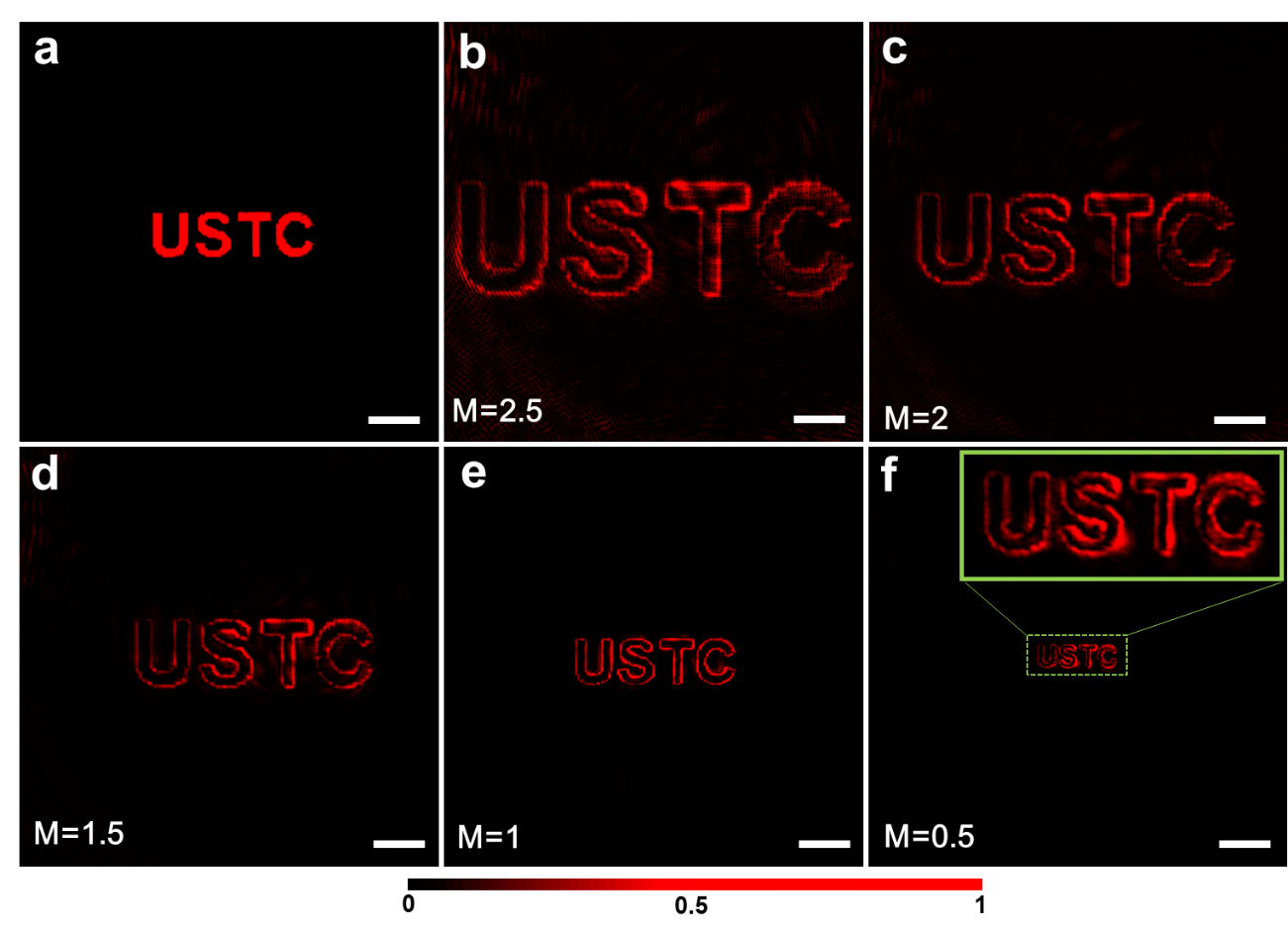


**Fig. S8 | (a)** Output energy intensity measured without meta-modulator with parameter *M=*1. **(b)-(g)** Output energy intensity of different magnification edge profiles. In our experiment, the meta-modulator has a detection accuracy of *w*_0_ =1.5 μm. Scalebars: 100 μm.

**10 | Edge detection of a larger-size object via doublet meta-imager**

**
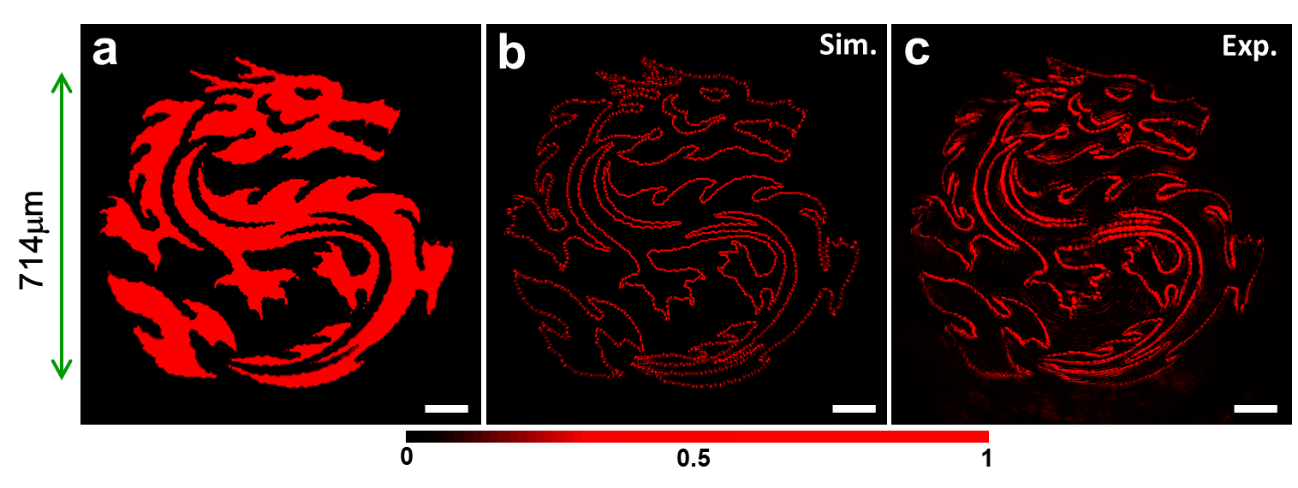
**

**Fig. S9 | Detecting the edges of a large object. (a)** The original object for edge detection. **(b-c)** Simulated **(b)** and experimental **(c)** edges by using the doublet meta-imager with the parameter *w*_0_ =1.5 μm, *M=*1 and *d*=*f*=2.5 mm. Scalebars: 100 μm.

To test the field-of-view, we put an amplitude object (dragon pattern etched through an opaque chromium film, see Fig. S9a) as the incidence of doublet meta-imager with the parameter *w*_0_ =1.5 μm, *M=*1 and *d*=*f*=2.5 mm. The simulated edges are shown in Fig. S9b, which has the good agreement with the experimental results in Fig. S9c. Both simulated and experimental edges can be observed clearly, which suggests a field-of-view better than 710 μm×710 μm at *M*=1. All the simulations are implemented in MATLAB by using the Rayleigh-Sommerfeld diffraction. The experimental setup used here is completely identical to the one in Fig. S7a. The discussions about the meta-imager including metasurface singlet and doublet can be found in more details in the following sections.

**11 | Edge enhancement via doublet meta-imager**

Image sharpening is used to enhance the contrast of a grayscale picture, so that the blurred image becomes clearer. Since Laplacian operator is an approximation of two-dimensional second-order differentiation, it is also used frequently to detect the edges directly, having the similar functionality with the edge-detection operator H_ED_ as reported in Fig. 2 of main text. Our edge-detection operator H_ED_ is the rigorous solution for two-dimensional one-order differentiation, which is the main difference from the Laplacian operator. In addition, the elements in the Laplacian operator have the real value, which can decrease the complexity of the computation compared with the H_ED_ operator and therefore is much preferred in traditional picture processing. Therefore, to demonstrate edge enhancement, we utilize the Laplacian operator H_L_ to extract the edges of objects instead of H_ED_, meanwhile adopting an additional operator H*_i_* to reload the original object. The Laplacian operator is

$H_{L}=\left[ \begin{matrix} -1 & -1 & -1 \\ -1 & 8 & -1 \\ -1 & -1 & -1 \end{matrix} \right]$,

and the operator *H_i_*

$H_{i}=\left[ \begin{matrix} 0 & 0 & 0 \\ 0 & 1 & 0 \\ 0 & 0 & 0 \end{matrix} \right]$.

Thus, our proposed operator for edge enhancement can be expressed as

$H_{EE}=H_{L}+{c*H}_{i}=\left[ \begin{matrix} -1 & -1 & -1 \\ -1 & 8+c & -1 \\ -1 & -1 & -1 \end{matrix} \right]$.

where the constant coefficient *c* is used to control the contrast between the extracted edge and the original object. In our demonstration, *c*=4 is used after a good balance between highlighting the edges and keeping the original object. The element pitch of $\mathcal{H}_{EE}$ is taken as *w*_0_=3 μm for the purpose of demonstration (see Fig. S10a). After the inverse Fourier transform, we obtain the amplitude (Fig. S10b) and phase (Fig. S10g) profiles of the meta-modulator. Experimentally, the corresponding meta-modulator (see Fig. S10c) with 3-level amplitude (1, 0.5 and 0.3) and 128-level phase has a saddle-shape amplitude transmission (cross-polarization part) profile after the discretization of the amplitude in terms of three different nanobricks (see SEM image in Fig. S10d), hereby leading to the expected transmission (equivalent to amplitude modulation) as depicted in Fig. S10e. The quantitative comparison of the experimental and simulated amplitude profiles in Fig. S10f reveals their good consistence and confirms the validity of the fabricated meta-modulator. In addition, the experimental phase encoded into the meta-modulator is retrieved and shown in Fig. S10h, demonstrating a linearly increasing phase expect the small jump caused by the propagation phase (Figs. S10i).

To validate its optical performance, a binary-amplitude object (Fig. S10j) is used as the input of this edge enhancement meta-imager. Fig. S10k shows the processed image by using our meta-imager, where the edges are enhanced meanwhile the original information is preserved. To illustrate it clearly, we plot the line (LL′ in Fig. S10l)-scanning intensity profiles across the edge in Fig. S10k, indicating good agreement in simulation and experiment.


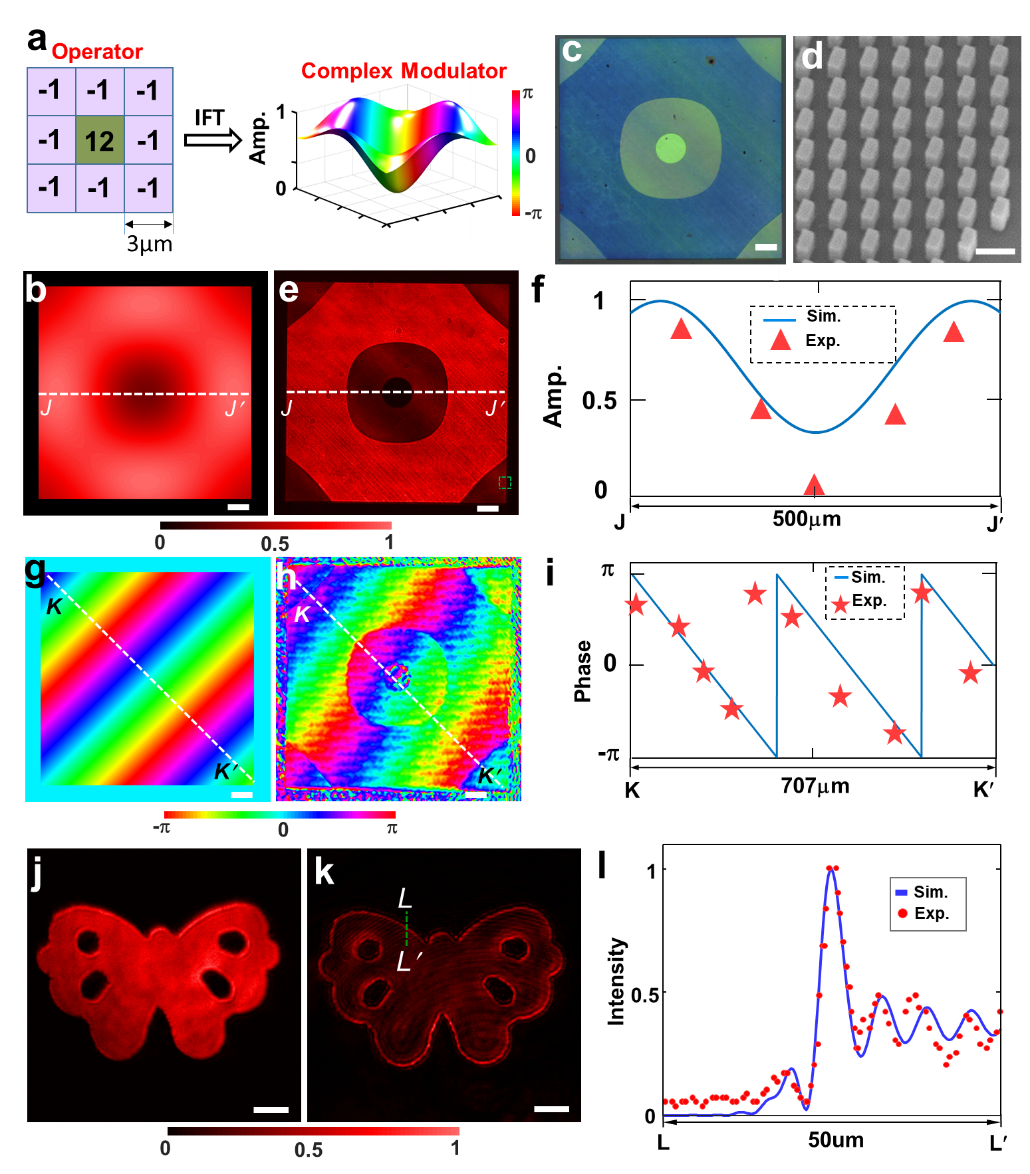


**Fig. S10 | Edge enhancement via doublet meta-imager. (a)** Proposed operator (left panel) and its related meta-modulator (right panel) for edge enhancement. In the panel of meta-modulator, the pseudo-color denotes the phase. **(b)** Amplitude profiles of meta-modulator. Scalebar: 50 μm. **(c)** Microscopic image of the fabricated meta-modulator. Scalebar: 50 μm. **(d)** SEM image of our fabricated modulator. Scalebar: 300nm. **(e)** Transmission (cross-polarization part) of the fabricated meta-modulator with the corresponding *w*_0_=1.5 μm under the circular-polarized illumination. Scalebar: 50 μm. **(g)** Simulated (curve) and experimental (triangles) line-scanning amplitude profiles along the line *JJ*′ (denoted in **(b)** and **(e)**). The experimental amplitude are obtained by using a square root of the transmission in **(e)**. **(g-h)** simulated phase **(g)** and retrieved **(h)** phase profiles from the experimental interference patterns for our fabricated meta-modulator. Scalebars: 50 μm. **(i)** Simulated (curve) and experimental (stars) phase profiles along the line *KK′* (denoted in **(g)** and **(h)**). **(j)** Original object obtained by using a single metalens without the modulator. Scalebar: 50 μm. **(k)** Processed image by our meta-imager with the modulator. The edge are enhanced and the original object is also maintained. Scalebar: 50 μm. **(l)** Normalized intensity profiles along the line LL′ (sketched in **(k)**). Both simulated (solid) and experiment (dashed) results have good agreement.

**12 | Size effect for the denoising meta-modulator**

Our denoising meta-modulator is designed with the accuracy of 4 μm, which could remove the defect with the size of 4 μm. In fact, such the designed meta-modulator with the fixed accuracy can also be used to remove the defects with different size. To illustrate it, we carry out the numerical simulations by applying our denoising meta-modulator with the given accuracy of 4 μm to remove the defects with the size ranging from 2 μm to 6 μm. The simulated results are shown in Figs. S11a-S11k, where the images by only a single metalens without the modulators are also provided as a control case. All the relative simulations are implemented by Rayleigh-Sommerfeld diffraction integrals. From all these simulations, one can observe the dark defects clearly if no modulator is employed. In comparison, when the denoising modulator is used, the defects can be suppressed efficiently, leading to the enhanced uniformity. To investigate it quantitatively, we compare the ratios of the intensity at the defect region to the ambient intensity in Fig. S11l, unveiling the enhanced uniformity that is related with the high ratio for the case with the denosing meta-modulator. Note that, when the size of the defect is smaller than the designed accuracy, the resulting uniformity is enhanced significantly because the defect is completely located within the accuracy range. For example, for the defects with the size of 2 μm, the corresponding uniformity after processed by our denoising modulator is nearly perfect because the ratio approaches 1 (see Fig. S11l), which means that both intensity profiles inside and outside the defects are equal with great uniformity. But, for the larger-size defects, the uniformity is less enhanced. Therefore, we can claim that the denoising meta-imager works well for the samples with the defect size smaller than the designed accuracy of the given meta-modulator.


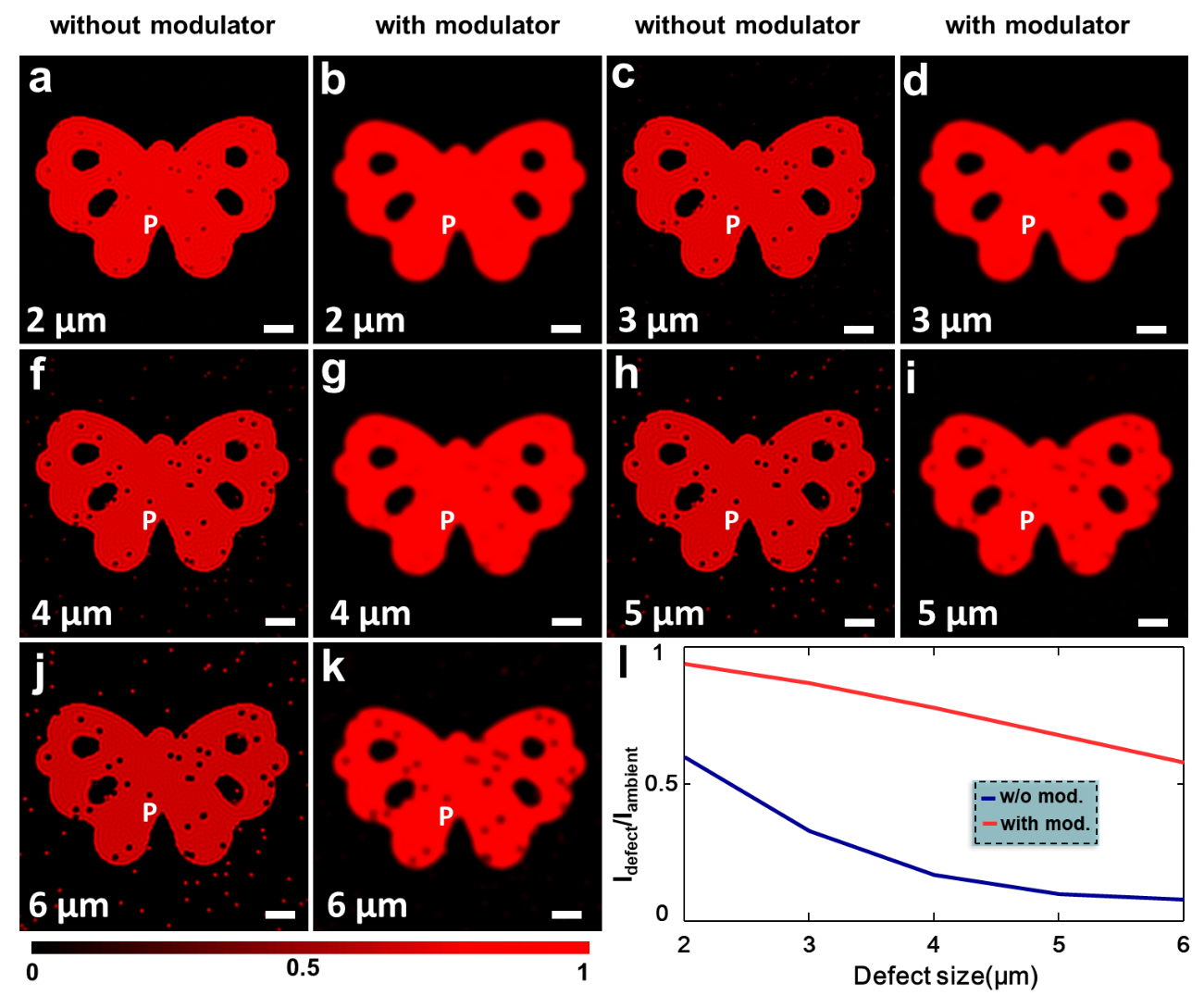


**Fig. S11 | Size effect of the denoising meta-imager. (a-k)** Simulated images by a single metalens (without meta-modulator) and the meta-imager (with meta-modulator) for the defects with different size. Scalebars: 40 μm. **(i)** Simulated intensity ratios (denoted ‘P’ in **(a-k)**). The data before (w/o mod.) and after (with mod.) using the meta-modulator is compared in details for the observation of the improved uniformity. The averaged intensity at the defect region (*i.e.*, 2^2^~6^2^ μm^2^) is labelled as *I*_defect_, while the averaged intensity at the surrounding region that has no defect is calculated as *I*_ambient_.

**13 | Field-of-view of our meta-imager**

According to Eq. (1) in main text and Eq. (S10) in the Supporting Information, the field-of-view is determined by the parabolic phase $k\frac{M}{2f}\cdot\frac{f-d}{l_{2}-d}r_{0}^{2}$, which leads to optical off-axis aberration. To illustrate the field-of-view for the meta-imagers with different *d*, we implement the numerical simulations to check the realistic imaging regions that can be taken as efficient field-of-view. Note that, although the simulations are done by using the edge-detection operation as an example, the results can be extended to other meta-imagers with different functionalities due to their identical working principles. Owing to the axisymmetric feature in the imaging systems, we employ a binary-amplitude concentric rings (Fig. S12a) with the same widths of 12.5 μm, which is chosen for a clear discrimination of the extracted edges and therefore determines the precision of our simulations. In our simulations, the parameters are fixed with *w*_0_ =1.75 μm, *M*=1, *f*=2.5 mm and λ=633nm, expect the only variable of *d* that ranges from *d*=0 to *d*=*f*. After using our meta-imager, the simulated intensity profiles at the image plane are shown in Figs. S12b-S12m, which denotes the efficient detection region with clear edges by addressing the parameter *d*.


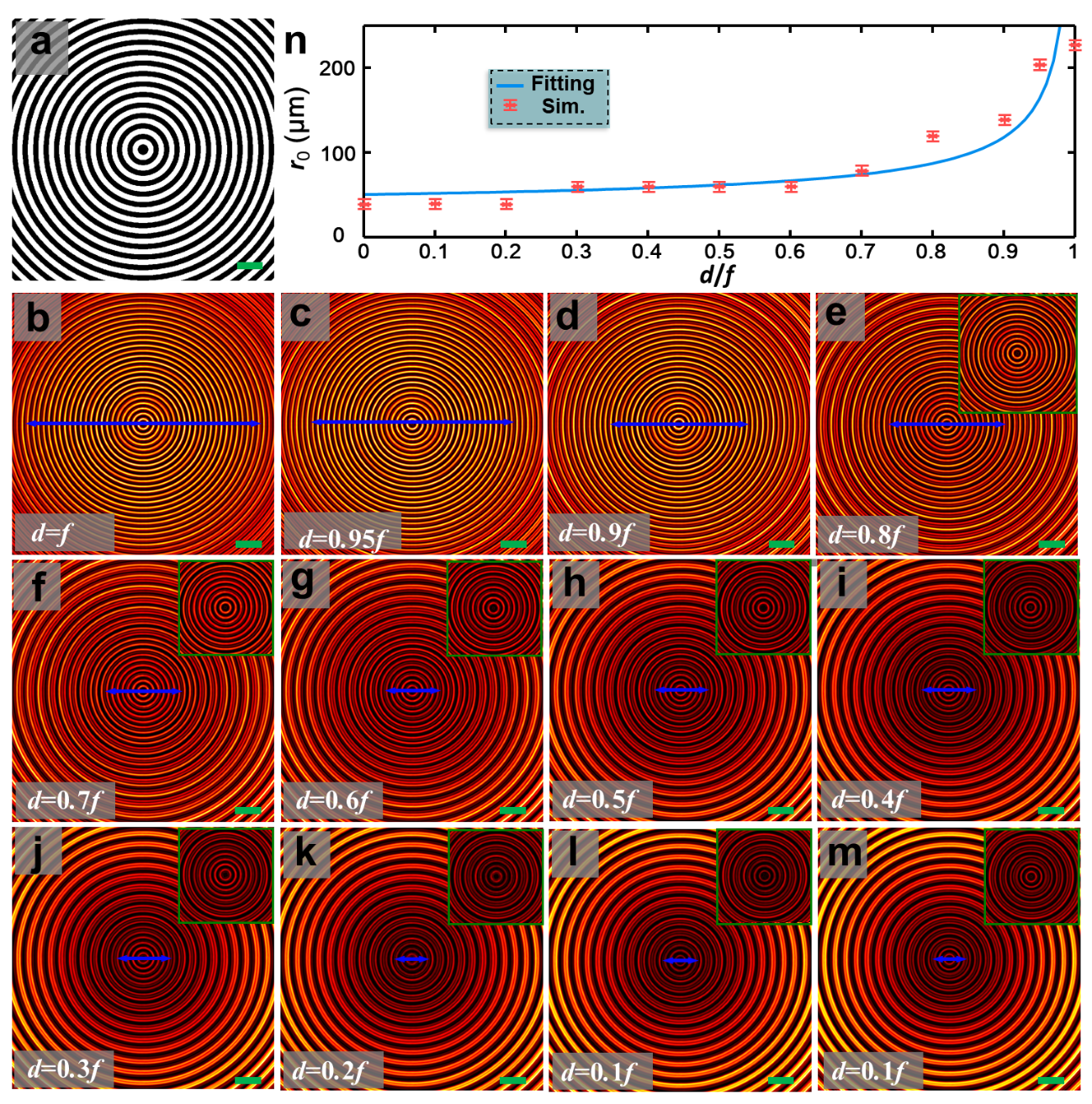


**Fig. S12. (a)** Input image wth an amplitude equally spaced rings object. Scalebar: 50 μm. **(b-m)** Simulated output intensity images using the meta-imager with parameter *w*_0_ =1.5 μm and *M*=1. The length of the blue arrows represent the field of view in **(n)-(m)**. The small image in the upper right corner is an enlarged view of the central area in **(e)-(m).** Scalebars: 50 μm. **(n)** Relationship between the *d*/*f* and the *r*_0_ when $k\frac{M}{2f}\cdot\frac{f-d}{l_{2}-d}r_{0}^{2}$=0.8π. The internal intensity between two adjacent edges is not higher than the average value of the two edge intensity peaks, which is considered to be within the field of view. Both simulated (dashed) and predicted (solid) results have good agreement. Considering the edge detection error, we provide the simulated *d/f*-dependent *r*_0_ profiles if the *r*_0_ is deviated by ±5 μm.

During the evaluation, we define the efficient detection region, where the ratio of the peak intensity between two neighboring edges to the averaged intensity of these two edges is smaller than 1/3. If the ratio is too large, the peak intensity between two neighboring edges is comparable to the intensity edges so that one cannot distinguish the edges. By following this definition, we derive the field-of-view at different *d*, which has been illustrated in Fig. S13n. It indicates an increasing field-of-view (in terms of *r*_0_) with the increment of *d* during the range of interest. To correlate these simulations with the parabolic phase, we carry out a fitting by using the phase $k\frac{M}{2f}\cdot\frac{f-d}{l_{2}-d}r_{0}^{2}=p$, where *p* is a constant. The good fitting is achieved at *p*=0.8π, where the relationship between *r*_0_ and *d* is illustrated by the solid curve in Fig. S12n. Both fitted and simulated results have good agreement with each other, implying that the field-of-view of our meta-imager can be evaluated by using *r*_0_^2^=$\frac{0.8\pi}{k}\frac{2f}{M}\cdot\frac{l_{2}-d}{f-d}$.

**14 | Spiral phase contrast microscopy**

Traditional optical microscopy can only image the high-contrast amplitude objects. Most biological samples such as cells are the phase-type objects, which cannot be observed clearly. In 1934, Zernike invented phase contrast microscopy (PCM) ^7^, where the binary (0-π) phase along one-dimensional direction is located at the Fourier plane of the imaging objective. Such a configuration works as one-dimensional differentiation of object so that the tiny phase variation in the biological sample can also be revealed. However, one-dimensional differentiation is not sufficient in optical picture processing. To realize two-dimensional differentiation, a spiral phase with an azimuthal-dependent wavefront is employed because such a spiral phase offers a 0-π binary phase along any radial direction ^8-10^, which leads to the isotropic optical differentiation in two dimensional directions. Thus, such a spiral-phase-based contrast microscopy is frequently used to extract the edges of the biological samples. Note that, because only phase modulation is provided in such a spiral phase plate, the detection accuracy (usually defined by the width of the detected edges) is not controllable ^11^, resulting in a low accuracy. In contrast, our proposed meta-imager has solved this problem by customizing the pixel size of the convolutional operator (i.e., the 3×3 matrix).

**15 | A brief introduction to convolutional neural network**

In order to show the importance of our work, we provide a brief introduction to convolutional neural network. Artificial neural network (ANN) is a mathematical or computational model that mimics biological neural networks (such as the central nervous system of animals, especially the brain) for realizing the artificial intelligent system. It contains a huge number of nodes (or neurons), each of which is connected with others. Each node is named as an activation function and each connection between two nodes is evaluated by a weighted network output that depends on the connecting way in the network. ANN can process information by adjusting the weight among these internal neurons, so as to realize the purpose of processing information. Convolutional neural network (CNN), as a feedforward neural network, has been proposed in 1990 ^12,13^ and widely accepted in 2012 ^14^ due to the excellent performance in image recognition. The CNN has the basic architecture consisting of input layer, convolutional layer and pooling layer (also known as sampling layer). The convolution layer and pooling layer (formed by the full connection layer and output layer) are generally selected in an alternate way, that is, a convolution layer is firstly connected to a pooling layer, which is then followed by another convolution layer in turn. In each neuron, the outputted feature of the processed graph in the convolutional layer is locally connected with the input, which is obtained through the weighted sum of the corresponding connection weight and the bias value.

Among these operations in CNN, the convolutional layer is the most time-consuming due to the convolutional operation. Convolution operations between a digital image and an operator (usually a 3×3 matrix) need three steps: i) spatial shift in two dimensions; ii) multiple product of matrix elements that are spatially overlayed between the image and the operator; iii) a summation of these products, hereby leading to the time-cost issue. In each layer, numerous convolutional operators with functionality-assigned kernels are implemented to extract the important features of objects for identification. Here, we introduce several frequently used convolution operators.

1) **Average filtering**. An average filter has the same value that is the reciprocal of the element number of the convolution kernel. It yields the average of its surrounding pixels. An average filter of 3×3 matrix is shown as follows:

$\left[ \begin{matrix} 1/9 & 1/9 & 1/9 \\ 1/9 & 1/9 & 1/9 \\ 1/9 & 1/9 & 1/9 \end{matrix} \right]$, (S13)

which is usually used for image blur/smoothing to eliminate the noise.

2) **Image sharpening**. To better distinguish an image, we usually need to highlight its details, which usually refer to the high-frequency part in the image. The convolution kernel for image sharpening can be taken as a kind of high-pass filter. An exemplified 3×3 matrix for image sharpening is shown by

$\left[ \begin{matrix} -1 & -1 & -1 \\ -1 & 9 & -1 \\ -1 & -1 & -1 \end{matrix} \right]$, (S14)

which outlines the difference between the central element and its surrounding. The large difference means a big change (frequency) around the element.

**3) First-order differentiation**. The edge of an object in an image offers the most important features that can help the recognition of the object. At the edge, the image grayscale of the object changes dramatically, resulting mathematically in a large derivative. Therefore, the directional derivative of grayscale in an image can highlight the edge of the object. Its corresponding operator is the Sobel operator :

$\left[ \begin{matrix} 1 & 0 & -1 \\ 2 & 0 & -2 \\ 1 & 0 & -1 \end{matrix} \right]$ , (S15)

which yields the horizontal edge (*i.e.*, *x* direction). Its transposed matrix stands for the *y*-direction differentiation operator. To obtain two-dimensional differentiation, one can refer to Eq. (3) in the main text, where two orthogonal derivative operators in *x* and *y* direction are encoded in the real and imagery parts, respectively. Such a configuration of two-dimensional derivative operators with complex values is easily realized in optics due to the parallel features.

**4) Laplace operator**. The Sobel operator introduced above is used to output one-dimensional differentiation of the image. The Laplace operator is used to realize the second-order derivative operation. Its typical form is expressed as:

$\left[ \begin{matrix} 1 & 1 & 1 \\ 1 & -8 & 1 \\ 1 & 1 & 1 \end{matrix} \right]$, (S16)

where is described as the difference between the central element and the surrounding pixel. In practical applications of digital convolutions, it usually is used to output the edge of an object.

**16 | A comparison among the reported all-optical convolutional approaches**

To give a detail review about the reported all-optical convolutional approaches, we list the important features in Table S1 by addressing different literatures. These approaches are categorized into three cases: Fourier spatial filtering, Green’s function and the meta-imager. The parameters such as wavelength, platforms, field of view, magnification, resolution, integration level and demonstrated functionalities are provided with the detailed value. It is worthy pointing out that the values of some parameters in the table are derived indirectly from the corresponding literatures. To give a fair comparison, we provide the field-of-view in units of wavelength because these all-optical convolutions operates at different wavelengths.

**Table S1 | A comparison between all-optical convolutional operations.**

| **Approaches** | **Literatures** | **Wavelength**  **(nm)** | **Platforms** | **Field of view** | **Magnification** | **Resolution** | **Integration**  **Level** | **Demonstrated Functionalities** | | | |
| --- | --- | --- | --- | --- | --- | --- | --- | --- | --- | --- | --- |
|  |  |  |  |  |  |  |  | **Edge detection** | **Differentiation** | **Edge**  **Enhancement** | **Denoi-sing** |
| Fourier spatial filtering | Silva et al.^15^ | 3000 | Transmissive metasurfaces | 10λ | 1 | Not reported | High | × | √ | × | × |
|  | Wang et al. ^16^ | 500 | On-chip slot arrays | 20λ | 1 | 0.5μm | High | × | √ | × | × |
|  | Zhou et al. ^17^ | 430~670 | SiO_2_ nano-gratings | Not reported | 1 | 2μm | Low | √ | √ | × | × |
|  | Zhou et al. ^18^ | 410~690 | SiO_2_ nano-gratings | 10256λ | 1 | Not reported | Low | √ | × | × | × |
|  | Zhou et al. ^19^ | 405 | SiO_2_ nano-gratings | 4740λ | 1 | Not reported | Low | √ | × | × | × |
|  | Pors et al. ^20^ | 800 | Reflective plasmonic metasurfaces | 5000λ | 1 | Not reported | Low | × | √ | × | × |
|  | Qiu et al.^21^ | 1064 | KTP nonlinear crystal | Not reported | 1 | Not reported | Low | √ | × | × | × |
|  | Huo et al. ^22^ | 480~630 | Dielectric geometric  metasurfaces | 1460λ | 1 | 3.11 µm. | Low | √ | × | × | × |
| Green’s function | Silva et al.^15^ | 3000 | Meta-transmit-array | 60λ | 1 | Not reported | High | × | √ | × | × |
|  | Zhu et al. ^23^ | 532 | Plasmonic film | 2125λ | 1 | 7.2μm | Low | × | √ | × | × |
|  | Cordaro et al.^24^ | 726, 750 | Si Nano-gratings | 480λ | 1 | Not reported | Low | √ | √ | × | × |
|  | Guo et al. ^25^ | 1400 | Photonic crystal slab | 35λ | 1 | 20μm | High | √ | × | × | × |
|  | Kwon et al.^26^ | 2.489×10^8^  (millimeter wave) | Split-ring resonators | 1314λ | 1 | Not reported | Low | √ | √ | × | × |
|  | Zhou et al.^27^ | 740~1280 | Si nano-pillars | 690λ | 1 | 4μm | High | √ | × | × | × |
|  | Davis et al. ^28^ | white light | Gold nanorods | 40λ | 1 | Not reported | High | × | √ | × | × |
|  | Kwon et al. ^29^ | 850 | Silicon metasurfaces | 400λ | 1.6, 1.98 | 2.76μm | High | × | √ | × | × |
|  | Zhu et al. ^30^ | 532~820 | Reflection of bulk prisms | 3195λ | 1 | 5μm | Low | √ | × | × | × |
|  | Zhu et al.^31^ | 532 | Reflection of bulk prisms | 940λ | 1 | 3.2μm | Low | × | √ | × | × |
|  | Andrei Komar et al.^32^ | 1400, 1570 | Si nanodisks metasurfaces | 286λ | 1 | 7µm | High | × | √ | × | × |
| Meta-imager | This work | 633 | Single-crystal silicon geometric metasurfaces | 1269λ | 0.5~2.5 | 4μm | High | √ | √ | √ | √ |

From this table, we can conclude that most of Fourier spatial filtering approaches have the main drawback of low integration, while most approaches about Green’s function has the limited functionalities. None of these two approaches can achieve both high integration and multiple functionalities simultaneously. Fortunately, our meta-imager approach offers a viable solution to high-integration and multi-functionality all-optical convolutions. Beyond this, our meta-imager also has a large field-of-view, tunable magnification, as well as high spatial resolution in convolutional processing. From the viewpoint of application, our meta-imager has bridged the gap between optical complex-amplitude elements and the required convolutional operators that have been frequently used in traditional picture processing and convolutional neural networks, hereby exhibiting the good compatibility with electronic convolutional operations.

**References**

[1]. Jeffrey, A., & Zwillinger, D. *Table of integrals, series, and products*, (Academic Press, **2007**).

[2]. Huang, K., Dong, Z., Mei, S., Zhang, L., Liu, Y., Liu, H., Zhu, H., Teng, J., Luk'yanchuk, B., Yang, J. K., & Qiu, C., Silicon multi‐meta‐holograms for the broadband visible light, *Laser Photonics Rev.* **10**, 500-509(2016).

[3]. Desiatov, B., Mazurski, N., Fainman, Y., & Levy, U., Polarization selective beam shaping using nanoscale dielectric metasurfaces, *Optics Express* **23**, 22611-22618(2015).

[4]. Huang, K., Qin, F., Hong Liu, Ye, H., Qiu, C. W., Hong, M., Luk'yanchuk, B., & Teng, J., Planar Diffractive Lenses: Fundamentals, Functionalities, and Applications, *Adv. Mater.* **30**, 1704556(2018).

[5]. Huang, K., Liu, H., Restuccia, S., Mehmood, M. Q., Mei, S., Giovannini, D., Danner, A., Padgett, M. J., Teng, J., & Qiu, C.-W., Spiniform phase-encoded metagratings entangling arbitrary rational-order orbital angular momentum, *Light: Sci. Appl.* **7**, 17156(2018).

[6]. Huang, K., Zhao, D., Tjiptoharsono, F., Chen, Y., Wong, C. P. Y., Tang, X., Yang, J. K. W., & Dong, Z., Bio-inspired Photonic Masquerade with Perturbative Metasurfaces, *ACS Nano* **14**, 7529-7537(2020).

[7]. Zernike, F., Diffraction theory of the knife-edge test and its improved form, the phase-contrast method”. *Roy. Astron. Soc.* **94**, 377(1934).

[8]. Davis, J. A., McNamara, D. E., Cottrell, D. M., & Campos, J., Image processing with the radial Hilbert transform: theory and experiments, *Optics Letters* **25**, 99-101(2000).

[9]. Fürhapter, S., Jesacher, A., Bernet, S., & Ritsch-Marte, M., Spiral phase contrast imaging in microscopy, *Optics Express* **13**, 689-694(2005).

[10]. Ritsch-Marte, M., Orbital angular momentum light in microscopy, *Philosophical Transactions of the Royal Society A: Mathematical, Physical and Engineering Sciences* **375**, 20150437(2017).

[11]. Maurer, C., Jesacher, A., Bernet, S., & Ritsch-Marte, M., What spatial light modulators can do for optical microscopy, *Laser Photonics Rev* **5**, 81-101(2010).

[12]. Lang, K. J., Waibel, A. H., & Hinton, G. E., A time-delay neural network architecture for isolated word recognition, *Neural networks* **3**, 23-43(1990).

[13]. LeCun, Y., Boser, B., Denker, J., Henderson, D., Howard, R., Hubbard, W., & Jackel, L., Handwritten digit recognition with a back-propagation network, *Advances in neural information processing systems* **2**(1989).

[14]. Krizhevsky, A., Sutskever, I., & Hinton, G. E., Imagenet classification with deep convolutional neural networks, *Advances in neural information processing systems* **25**, 1097-1105(2012).

[15]. Silva, A., Monticone, F., Castaldi, G., Galdi, V., Alù, A., & Engheta, N., Performing Mathematical Operations with Metamaterials, *Science* **343**, 160-163(2014).

[16]. Wang, Z., Li, T., Soman, A., Mao, D., Kananen, T., & Gu, T., On-chip wavefront shaping with dielectric metasurface, *Nature Communications* **10**, 3547(2019).

[17]. Zhou, J., Qian, H., Chen, C.-F., Zhao, J., Li, G., Wu, Q., Luo, H., Wen, S., & Liu, Z., Optical edge detection based on high-efficiency dielectric metasurface, *Proceedings of the National Academy of Sciences* **116**, 11137-11140(2019).

[18]. Zhou, J., Qian, H., Zhao, J., Tang, M., Wu, Q., Lei, M., Luo, H., Wen, S., Chen, S., & Liu, Z., Two-dimensional optical spatial differentiation and high-contrast imaging, *Natl. Sci. Rev*, DOI:10.1093/nsr/nwaa1176(2020).

[19]. Zhou, J., Liu, S., Qian, H., Li, Y., Luo, H., Wen, S., Zhou, Z., Guo, G., Shi, B., & Liu, Z., Metasurface enabled quantum edge detection, *Science Advances* **6**, eabc4385(2020).

[20]. Pors, A., Nielsen, M. G., & Bozhevolnyi, S. I., Analog Computing Using Reflective Plasmonic Metasurfaces, *Nano Letters* **15**, 791-797(2015).

[21]. Qiu, X., Li, F., Zhang, W., Zhu, Z., & Chen, L., Spiral phase contrast imaging in nonlinear optics: seeing phase objects using invisible illumination, *Optica* **5**, 208-212(2018).

[22]. Huo, P., Zhang, C., Zhu, W., Liu, M., Zhang, S., Zhang, S., Chen, L., Lezec, H. J., Agrawal, A., Lu, Y., & Xu, T., Photonic Spin-Multiplexing Metasurface for Switchable Spiral Phase Contrast Imaging, *Nano Letters* **20**, 2791-2798(2020).

[23]. Zhu, T., Zhou, Y., Lou, Y., Ye, H., Qiu, M., Ruan, Z., & Fan, S., Plasmonic computing of spatial differentiation, *Nature Communications* **8**, 15391(2017).

[24]. Cordaro, A., Kwon, H., Sounas, D., Koenderink, A. F., Alù, A., & Polman, A., High-index dielectric metasurfaces performing mathematical operations, *Nano letters* **19**, 8418-8423(2019).

[25]. Guo, C., Xiao, M., Minkov, M., Shi, Y., & Fan, S., Photonic crystal slab Laplace operator for image differentiation, *Optica* **5**, 251-256(2018).

[26]. Kwon, H., Sounas, D., Cordaro, A., Polman, A., & Alù, A., Nonlocal Metasurfaces for Optical Signal Processing, *Phys Rev Lett* **121**, 173004(2018).

[27]. Zhou, Y., Zheng, H., Kravchenko, I. I., & Valentine, J., Flat optics for image differentiation, *Nature Photonics* **14**, 316-323(2020).

[28]. Davis, T. J., Eftekhari, F., Gómez, D. E., & Roberts, A., Metasurfaces with Asymmetric Optical Transfer Functions for Optical Signal Processing, *Phys Rev Lett* **123**, 013901(2019).

[29]. Kwon, H., Arbabi, E., Kamali, S. M., Faraji-Dana, M., & Faraon, A., Single-shot quantitative phase gradient microscopy using a system of multifunctional metasurfaces, *Nature Photonics* **14**, 109–114(2020).

[30]. Zhu, T., Guo, C., Huang, J., Wang, H., Orenstein, M., Ruan, Z., & Fan, S., Topological optical differentiator, *Nature Communications* **12**, 680(2021).

[31]. Zhu, T., Lou, Y., Zhou, Y., Zhang, J., Huang, J., Li, Y., Luo, H., Wen, S., Zhu, S., Gong, Q., Qiu, M., & Ruan, Z., Generalized Spatial Differentiation from the Spin Hall Effect of Light and Its Application in Image Processing of Edge Detection, *Physical Review Applied* **11**, 034043(2019).

[32]. Komar, A., Aoni, R. A., Xu, L., Rahmani, M., Miroshnichenko, A. E., & Neshev, D. N., Edge Detection with Mie-Resonant Dielectric Metasurfaces, *ACS Photonics* **8**, 864-871(2021).
